# Documenting the occurrence of microbial carbonate deposits in Puerto Rico

**DOI:** 10.1038/s41598-026-47709-x

**Published:** 2026-04-13

**Authors:** Bryan J. Rodríguez-Colón, Wilson R. Ramírez-Martínez, Aliyah M. Chabrier-Alpi, Carlos Ríos-Velázquez, Noraida Martínez-Rivera, Gregory S. Baker, Yasawantha Hiripitiyage, Belinda S. M. Sturm, Jennifer A. Roberts

**Affiliations:** 1 Department of Geology, University of Kansas, Lawrence, KS 66045 USA; 2 Department of Geology, University of Puerto Rico–Mayagüez, Mayagüez, PR 00681 USA; 3 Department of Marine Sciences, University of Puerto Rico–Mayagüez, Mayagüez, PR 00681 USA; 4 Department of Biology, University of Puerto Rico–Mayagüez, Mayagüez, PR 00681 USA; 5 Microscopy and Analytical Imaging Laboratory, University of Kansas, Lawrence, KS 66045 USA; 6 Geoscience Program, Colorado Mesa University, Grand Junction, CO 81501 USA; 7 Department of Civil, Environmental & Architectural Engineering, University of Kansas, Lawrence, KS 66045 USA

**Keywords:** Microbial Carbonates, Microbial Mats, Microbialites, Puerto Rico, Guánica, Mg-carbonates, Biogeochemistry, Ecology, Ecology, Environmental sciences, Microbiology, Ocean sciences, Solid Earth sciences

## Abstract

Microbial carbonate deposits, including microbial mats and microbialites, record coupled biological, geochemical, and sedimentary processes, providing insight into biosedimentary dynamics through Earth’s history and serving as analogs for ancient and potentially extraterrestrial environments. This study presents the first documentation of lithifying microbial systems in Puerto Rico, identified within three proximal coastal lagoons (Salineta, Vernales, and Providencia) along the southwestern coast of the Guánica municipality. These lagoons define a continuum of lithification, ranging from non-lithifying hypersaline mats to fully lithified microbialite deposits. An integrated field, geochemical, petrographic, and microbial ecological approach was used to characterize deposit morphology, mineralogy, and microbial community structure. At Salineta, elevated salinity, low pH, and evaporitic conditions correspond to halite- and gypsum-dominated mats hosting halophilic Archaea and anoxygenic phototrophs, with minimal carbonate precipitation. In Vernales, carbonate precipitates closely associated with microbial filaments and EPS indicate enhanced organomineralization. Providencia hosts extensive lithified deposits displaying composite stromatolitic and thrombolitic fabrics, localized occurrences of high-Mg carbonate phases, and microbial communities enriched in sulfate-reducing bacteria relative to the other lagoons. Collectively, variations in hydrology, salinity, substrate, and microbial community composition correspond to differing degrees of lithification across a small coastal region, establishing southwestern Puerto Rico as a natural laboratory for investigating microbialite formation and biosignature preservation in coastal carbonate systems.

**Supplementary Information:**

The online version contains supplementary material available at 10.1038/s41598-026-47709-x.

## Introduction

Modern microbial carbonate mineral deposits, including microbial mats and microbialites, provide valuable insights into the geobiological evolution of Earth^1^. Microbialites, including laminated stromatolites and clotted thrombolites, form through the lithification of microbial mats via sediment trapping, binding, and in situ organomineralization (i.e., microbially induced mineralization)^2,3^. Although microbialites were widespread during the Precambrian, with the oldest examples dating to ca. 3.5 Ga, their abundance declined markedly through the Phanerozoic^1,4^. Today, unlithified microbial mats (vertically laminated biofilms composed of diverse microbial communities) are common in shallow aquatic environments globally, yet only a subset undergoes sustained lithification, making modern microbialites comparatively rare^5^. This disparity highlights a fundamental and unresolved question: why do some microbial mats lithify while others remain unlithified under broadly similar environmental conditions? Because modern microbialites preserve coupled biological, mineralogical, and geochemical signatures, they also serve as valuable analogs for interpreting ancient biosedimentary systems and potential biosignatures in extraterrestrial settings, including Mars (e.g.^6,7^) .

Previous research from microbialite-forming environments has improved understanding of mechanisms governing organomineralization within these systems^8–16^. These processes reflect complex interactions among microbial community composition, metabolic activity, extracellular polymeric substances (EPS), and physicochemical conditions such as salinity, pH, mineral saturation state, and water chemistry, as well as broader environmental influences operating across geomorphic and sedimentary contexts^3^. Despite these advances, the relative importance and interaction of microbial processes, aqueous geochemistry, and environmental forcing remain incompletely resolved. Continued characterization of lithifying microbial systems, particularly in new or understudied settings, is therefore necessary to better understand why some microbial mats undergo lithification while others remain unlithified.

Several microbial carbonate environments have been studied within coastal lagoons across the circum-Caribbean region, including sites in the Bahamas (e.g., Storr Lake, San Salvador Island, Big Pond, Eleuthera Island, and Windsor Point Salt Pond, Crooked-Acklins Platform)^8,17,18^, Venezuela (e.g., Laguna Pirata, Los Roques Archipelago)^19^, Cuba (e.g., Cayo Cocos and Sabana-Camagüey Archipelago)^20,21^, and Bonaire (e.g., GotoMeer Basin)^22^. In Puerto Rico, previous studies have focused on non-lithifying benthic and ephemeral microbial mats in hypersaline lagoons in Cabo Rojo^23,24^. However, actively lithifying microbial mats and microbialite deposits have not previously been described from other regions of the island.

This study documents, for the first time, the occurrence, internal structure, mineralogy, and microbial community composition of lithifying microbial deposits in Puerto Rico. This work focuses on three coastal lagoons in southwest Guánica Municipality: Salinas Vernales (characterized by calcifying microbial mats), Salinas Salineta (where microbial mats show minimal to no lithification), and Laguna Providencia (hosting fully developed microbialites). Together, these lagoons represent a spectrum of lithification states and composition, offering a valuable opportunity to examine the environmental and microbial factors associated with microbial mat lithification and microbialite development in dynamic coastal settings.

## Geologic Setting and Field Site Description

**Fig. 1:**
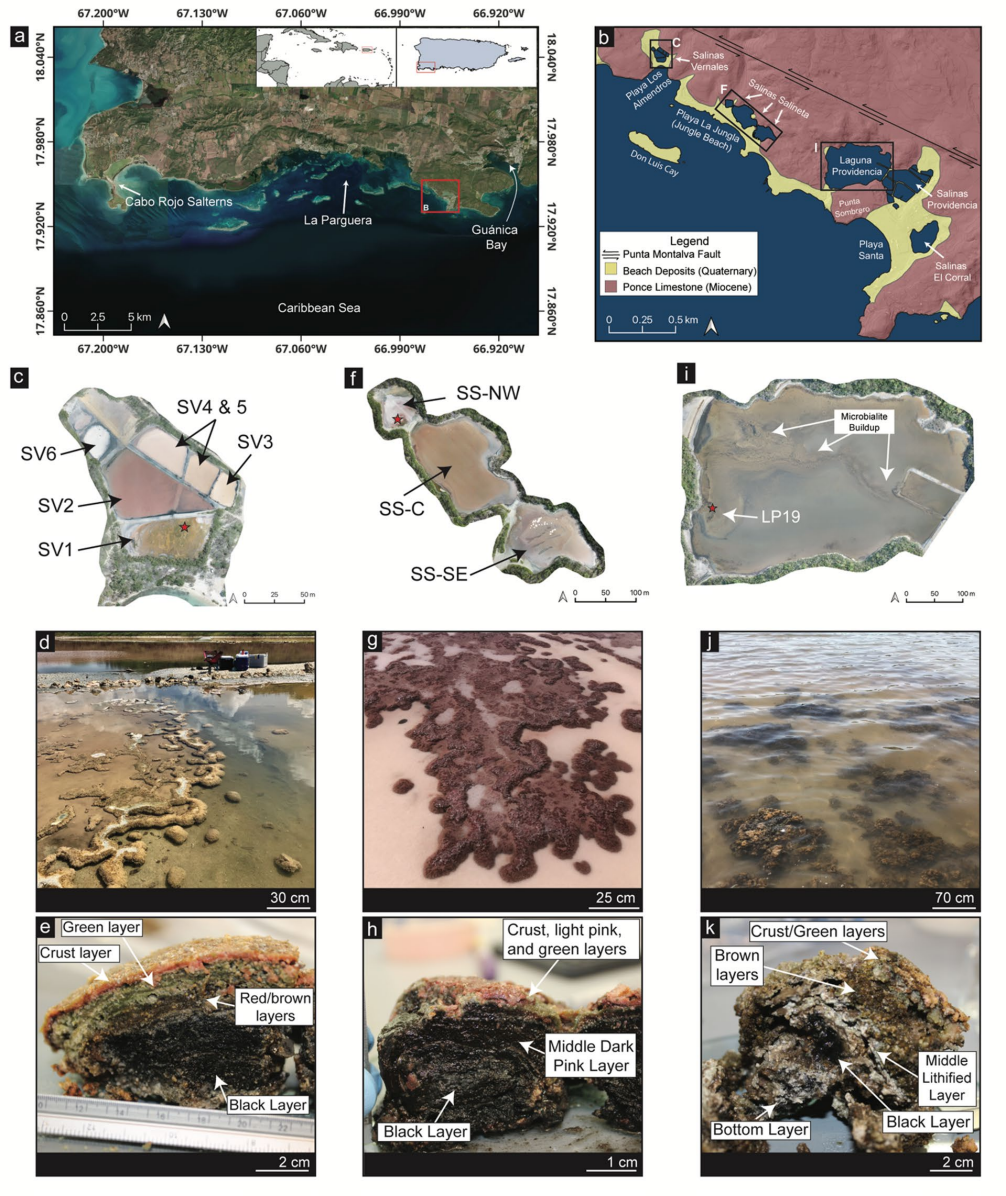
**Field site location of microbial deposits in Guánica**,** Puerto Rico. (a)** Satellite image showing the location of the study area in the Guánica region. **(b)** Simplified geologic map of the southwest coast of Guánica, highlighting the studied lagoons. **(c-e)** Salinas Vernales (17.95072° N, 66.97125° W): **(c)** drone image taken in 2019 showing the subdivided crystallizer ponds in Salinas Vernales (SV1-SV6); **(d)** field photograph of calcifying microbial mats from the southern pond (named SV1), with field station located in the stoned dike walkway; **(e)** representative sample collected and dissected for analysis. **(f-h)** Salinas Salineta (17.94663° N, 66.96545° W): **(f)** drone image of the three interconnected ponds in Salineta from 2019; **(g)** field photograph of pink halophilic microbial mats from the northwestern pond (SS-NW); **(h)** representative mat sample collected and dissected for analysis. **(i-k)** Laguna Providencia (17.94309° N, 66.95929° W): **(i)** drone image of the lagoon from 2019 showing the location of the microbialite buildup; **(j)** field photograph from the western sector of the lagoon where a sample (named LP19) was collected; **(k)** representative microbialite sample LP19 dissected for petrographic and mineralogical analysis. The studied lagoons represent hydrologically independent hypersaline basins within the same coastal salina complex and are not directly connected by surface water flow. *Image in panel (a) was generated using Q-GIS (v. 3.34.13-Prizren;* https://qgis.org*) with the ArcGIS World Imagery basemap. * *Source: Esri*,* DigitalGlobe*,* GeoEye*,* i-cubed*,* USDA FSA*,* USGS*,* AEX*,* Getmapping*,* Aerogrid*,* IGN*,* IGP*,* swisstopo*,* and the GIS User Community. Additional vector data include Natural Earth (1:10 m) and administrative boundaries from the Puerto Rico GIS portal (*https://gis.pr.gov*). Panels (c*,* f*,* and i) correspond to drone-derived orthomosaic imagery (see Methods for acquisition and processing details). Images in panels (d and j) were taken by Gregory Baker. Images in panels (g and e*,* h and k) were taken by Bryan Rodríguez-Colón.*

The study sites are three coastal lagoons near the town of Guánica, along the southwestern coast of Puerto Rico (Fig. 1a). The bedrock surrounding the lagoons is composed primarily of a Miocene-age unit named Ponce Limestone, a bioclastic packstone-wackestone unit that is partially dolomitized^25,26^(Fig. 1b). Dolomitization in Ponce Limestone is dated to ca. 10.21-6.83 Ma^26^, with dolomite-rich outcrops occurring immediately adjacent to the lagoon margins. Active tectonism has been documented in the region via the Punta Montalva fault (Fig. 1b), a left-lateral strike-slip fault system (trending ca. 110˚-115˚) that passes ca. 150-200 m north of the lagoons, with notable activity during a 6.4 M_w_ seismic event in January 2020^27,28^. Quaternary beach deposits isolating the lagoons from the Caribbean Sea are composed of modern coral and calcareous algal bioclasts (e.g., *Halimeda spp.*) and lithoclasts of Ponce Limestone^25^. The studied lagoons have mesosaline to hypersaline conditions most of the year and have been historically important for salt production in the area, due to relatively low annual rainfall in the southwest of Puerto Rico, ranging from 600 to 1,000 mm, and mean temperatures exceeding 26 °C^29,30^.

Salinas Vernales, the smallest lagoonal system (ca. 15,330 m²), comprises small, abandoned artificial solar salterns (ca. 0.2-0.35 m depth) adjacent to Playa de Los Almendros (Fig. 1b-c). Construction of the salterns is evidenced by subdivided crystallizer ponds, stoned dike walkways, and anthropogenic debris in the area (Fig. 1c-d; Supplementary Fig. S1a online). It is unclear whether a natural pond existed prior to the development of the salterns; however, historical aerial imagery shows that the southernmost pond (hereafter SV1) did not exist before the 1930s. This lagoonal system hosts localized calcifying microbial mats within one of the crystallizer ponds (SV1; Fig. 1c-d). Salinas Salineta is an intermediate-sized natural hypersaline system (ca. 41,380 m²) with minimal human modification (Fig. 1f). Historical documentation shows, however, that this system was also used for salt production, although in a lower capacity than the others from the area. This system comprises three interconnected ponds, positioned between Salinas Vernales and Laguna Providencia, and adjacent to Playa La Jungla (Fig. 1b). Within this system, unlithified microbial mats were observed across all three ponds. Laguna Providencia is the largest (ca. 0.25 km²), deepest lagoon (ca. 1.2-1.3 m depth south, ca. 0.8 m north) in the study area (Fig. 1i). The lagoon contains several anthropogenic modifications, including an artificial canal cut along the southwestern margin (ca. 150 m long) constructed between the late 19th and early 20th centuries, and abandoned solar salterns along the eastern side near Playa Santa neighborhood (Fig. 1b). This lagoon hosts spatially extensive and lithified microbialite deposits.

## Results

### Physicochemical Field Measurements, Distribution, and Macro Morphology of Microbial Deposits

*Salinas Vernales.* Calcifying microbial mats in Salinas Vernales were restricted to SV1, which is bordered by mangroves and covers ca. 1,600 m² of the lagoonal system (Fig. 1c; Supplementary Fig. S1a online). Preliminary aqueous measurements from SV1 indicate slightly basic conditions (pH ca. 8.3) with low salinity (ca. 25.4 ppt) and conductivity (ca. 39.4 mS), contrasting with higher salinity (ca. 78.5 ppt) and lower pH (ca. 6.8) in the adjacent northern pond (hereafter SV2), despite similar alkalinity values (Table 1). Microbial deposits in SV1 occur as isolated hemispheroidal mounds, coalescent mound clusters, and laterally continuous crusts up to ca. 15 m in length (Fig. 1d; Supplementary Fig. S1a online). Most deposits preferentially developed on hardened calcrete substrates, whereas areas lacking firm substrate were dominated by organic-rich ooze and lacked microbial mats. Polygonal and flat microbial mats occurred locally near pond margins, including zones with small pinnacle-like protrusions (Supplementary Fig. S1e online). In contrast, adjacent crystallizer ponds to the north (SV2-SV6) hosted only sparse, desiccated biofilms and lacked comparable deposits. A representative mat from the eastern margin of SV1 (SV1-E) was selected for petrographic, microscopic, and microbial diversity analyses (Fig. 1e).

**Table 1:** Physicochemical data collected across the lagoons.

Lagoon	Sample Name	pH	Temperature (˚C)	Specific Conductivity (mS)	Salinity (ppt)	Alkalinity (meq L^− 1^)
Salinas Vernales (SV1-SV2)	SV1	8.34	30.4	39.4	25.4	3.1
	SV2	6.78	34.4	111.3	78.5	3.1
Salineta (SS)	SS-NW	5.32	34.5	110.3	77.8	3.2
Providencia (LP)	LP19	7.33	32.3	80.6	55.3	2.4
	LP19-Flow	7.1	37.2	30.6	19.3	3.9

*Salinas Salineta.* The northwestern pond (hereafter SS-NW) hosted the most extensive microbial mat development within Salinas Salineta, with pink-pigmented mats forming atop evaporitic mineral crusts across ca. 3,750 m² (Fig. 1f-g; Supplementary Fig. S2a-c online). Waters in SS-NW were strongly saline and slightly acidic (salinity ca. 77.8 ppt; pH ca. 5.3; Table 1). Although petrographic analyses were not conducted on these deposits, SS-NW mats were analyzed for microbial community composition. Other ponds within Salinas Salineta contained only sparse or discontinuous microbial mats, including small chip-like mats on shell fragments (Supplementary Fig. S2 online) and mats enriched in detrital *Halimeda spp.* bioclasts.

*Laguna Providencia.* In Laguna Providencia, microbialites form a west-east-trending buildup extending ca. 350 m across the central sector of the western lagoon (Fig. 1i; Supplementary Fig. S3a online), with only sparse, small microbialites occurring along limited portions of the northern and southern shores. Surface waters exhibited near-neutral to slightly basic pH (ca. 7.1-7.3), moderate salinity (ca. 55 ppt), and low alkalinity (ca. 2.4 meq L⁻¹), whereas a localized inflow from the Ponce Limestone showed lower salinity (ca. 19 ppt) and higher alkalinity (ca. 3.9 meq L⁻¹; Table 1).

Drone-derived orthomosaics and field observations identify two dominant microbialite growth forms within the buildup: laterally continuous clustered microbialite accumulations (ca. 1-2 m) and more isolated mound-like structures (Supplementary Fig. S3b-c, e-f online). Clustered forms are more prevalent toward the western portion of the buildup, whereas isolated structures dominate toward the eastern margin and former crystallizer zones. Microbialite heads decrease in size from west to east and locally form linear to curvilinear alignments several meters in length along the southern portion of the buildup (Supplementary Fig. S3d online). Preservation ranges from partially lithified to sub-fossilized structures, with some microbialites supporting active pustular mats at their upper surfaces (Fig. 1k; Supplementary Fig. S4a). A representative microbialite from the western buildup (hereafter LP19) was selected for petrographic, mineralogical, and microbial analyses.

## Internal Structure, Microfabrics, and Mineralogy

**Fig. 2:**
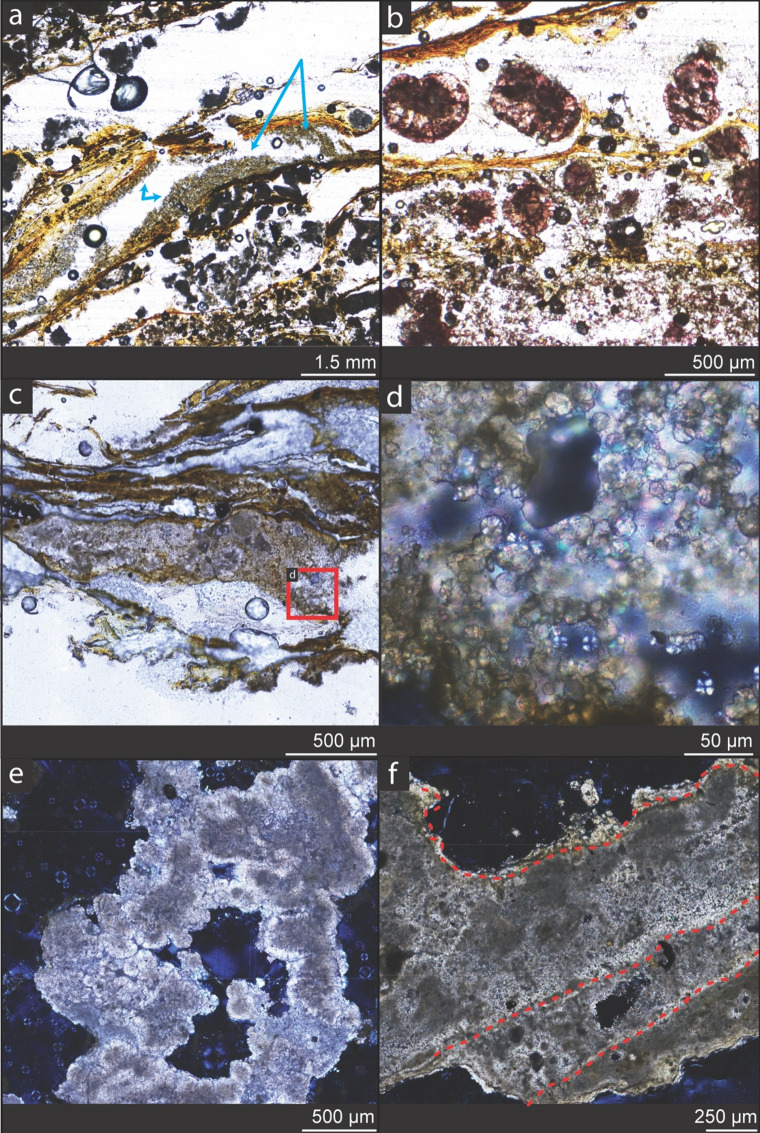
**Petrographic features of calcifying microbial mats in Salinas Vernales and microbialites from Laguna Providencia. (a-b)** Transmitted-light micrographs showing laminated microbial mat layers in Salinas Vernales (SV1) with micritic bands (light blue arrows) and interbedded organic-rich zones. Peloidal micrite and spherulitic grains **(b)** occur within EPS-rich laminae. The section shown in **(b)** was stained with Alizarin Red S and potassium ferricyanide to differentiate carbonate phases, showing red to pink tones in Mg-calcite bands and unstained aragonitic regions. **(c-d)** Detail of laminated mat region **(c)** and close-up **(d)** from active microbial mats in Laguna Providencia (LP) under cross-polarized light showing microbially mediated spherulitic aggregates nucleating within the EPS matrix. **(e-f)** Cross-polarized images from LP showing thrombolitic mesoclots composed of clustered spherulitic micro-peloids surrounded by micritic and acicular overgrowths (e), and alternating stromatolitic laminae outlined by red dashed lines (f).

**Fig. 3:**
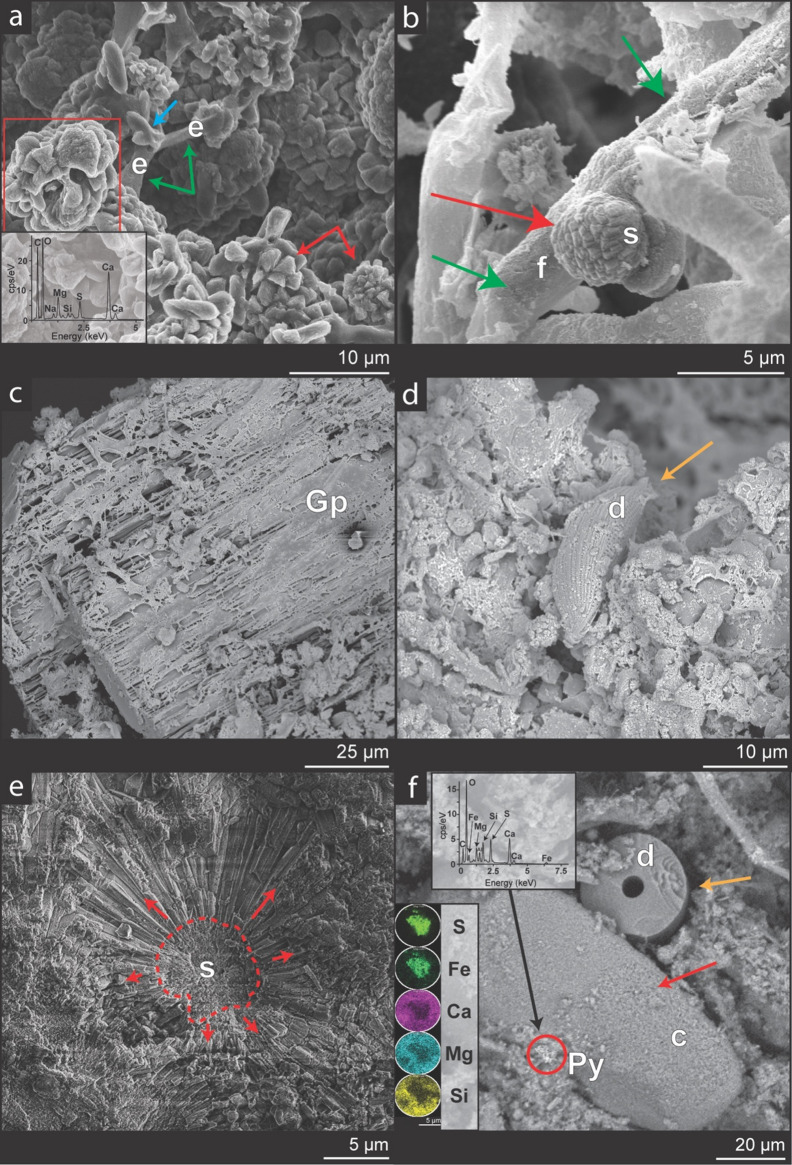
**Scanning electron microscopy (SEM) images showing microfabrics and mineral associations in microbial deposits. (a)** SEM micrographs of calcifying microbial mats in SV1E showing grains directly associated with EPS (letter e, green arrow). Some of the crystals show intertwined growth (blue arrow), while others have trigonal morphologies (red arrows). Insets in (a) display representative EDS spectra confirming Ca-Mg carbonate composition of these precipitates. **(b)** Small spherulite (letter s) directly growing from a filamentous microbial structure (letter f, green arrows) **(c-d)** Within the non-lithifying hypersaline mat in SS-NW showing euhedral gypsum crystals (letters Gp) embedded within the mat matrix **(c)** and **(d)** diatom (letter d; orange arrow) frustules coated by fine detrital and organic material. **(e-f)** Across the clustered thrombolitic microbialites fabrics from LP, spherulitic structures (letter s) with radiating aragonite needles were observed (red arrows). **(e)** Framboidal pyrite (letters Py, red circle), large carbonate grains (letter c, red arrow), and a putative diatom fragments (letter d, orange arrow) within micritic carbonate matrices. Elemental maps in (f) confirm the spatial association of S and Fe with the pyrite.

**Fig. 4:**
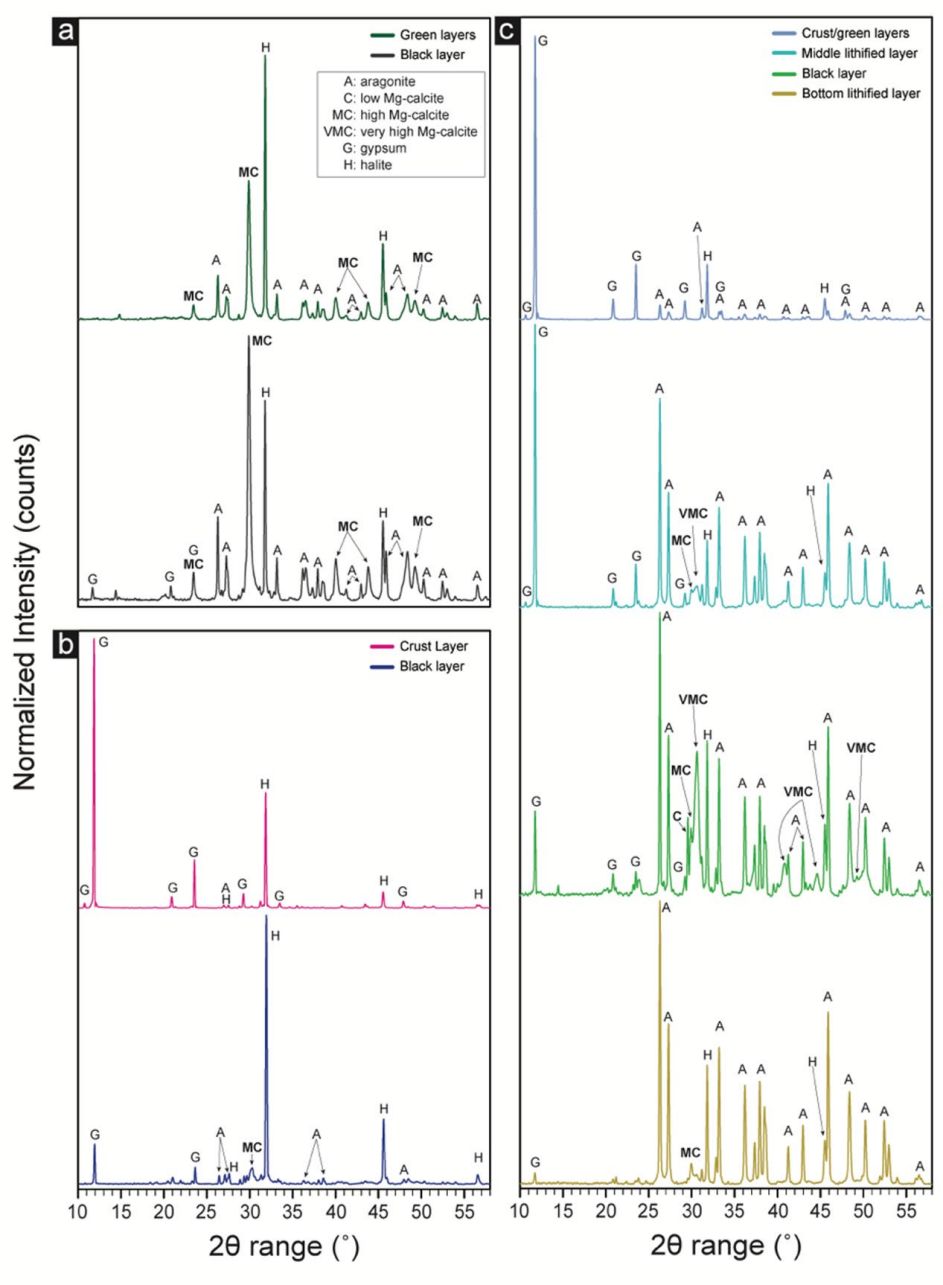
**X-ray diffraction (XRD) patterns of microbial mat and microbialite layers from southwestern Puerto Rico. (a)** In SV1-E, green and black mat layers showing dominant aragonite (A) and high-Mg calcite (MC) with minor gypsum (G) and halite (H). **(b)** Minerology of microbial mat crust and black layer from SS-NW was dominated by gypsum (G) and halite (H), with subordinate aragonite (A) and traces of high-Mg calcite (MC) in the black layers. **(c)** In LP19, the crust, intermediate, and basal layers show progressive mineralogical differentiation from aragonite-rich crusts to Mg-enriched carbonate phases in deeper zones. Very high-Mg calcite (VHMC) peaks occur exclusively in the black layers and were not detected in the bottom layers.

*Salinas Vernales (SV1-E).* Microbial mat from Salinas Vernales (SV1-E) exhibits clear vertical stratification, with thin pigmented surface layers grading into darker basal zones (Fig. 1e; Supplementary Fig. S1d, f). The mat fabric consists of alternating micritic and biofilm-rich laminae (Fig. 2a-b), locally incorporating micritic peloids and spherulitic grains (ca. 50-400 μm) with micritic nuclei and fibrous to botryoidal carbonate overgrowths. Sparse detrital components, including *Halimeda spp.* fragments and bivalve material, are present but volumetrically minor (Supplementary Fig. S5a online). SEM imaging shows abundant microbial filaments embedded within EPS matrices, closely associated with nanogranular to subhedral micritic precipitates, including small rhombohedral and trigonal aggregates attached directly to filaments (Fig. 3a-b; Supplementary Fig. S6a-b online). Larger spherulitic grains display fibrous internal textures and radial acicular growth. XRD analyses indicate that SV1-E mats are dominated by aragonite and high-Mg calcite (ca. 16.6 mol% MgCO₃), with Mg content increasing with depth (Fig. 4a).

*Salinas Salineta (SS-NW).* Microbial mats from Salinas Salineta display laminated fabrics broadly similar to those observed in SV1, but are distinguished by the presence of prominent pink-pigmented layers above and below the crust and a lack of carbonate lithification (Fig. 1g-h). No carbonate precipitates were observed within these mats; instead, evaporitic minerals dominate, including gypsum and a thick halite crust that serves as the primary substrate (Fig. 1g; Supplementary Fig. S2b). SEM observations reveal embedded gypsum crystals, pennate diatom frustules, and dispersed microbial filaments within the mat matrix (Fig. 3c-d). XRD analyses confirm gypsum and halite as the dominant mineral phases, with only minor aragonite and high-Mg calcite (ca. 27.1 mol% MgCO₃) detected in basal layers (Fig. 4b).

*Laguna Providencia (LP19).* The Laguna Providencia microbialite displays composite fabrics and extensive calcification. At the surface, pustular microbial mats form a thin crust composed of pigmented layers overlying a darker organic-rich zone, which in turn overlies lithified interior intervals and a basal layer (Fig. 1k). Sub-fossilized portions are dominated by thrombolitic textures in upper regions, characterized by clustered mesoclots, whereas deeper intervals contain irregular stromatolitic laminae (Supplementary Fig. S4 online).

**Fig. 5:**
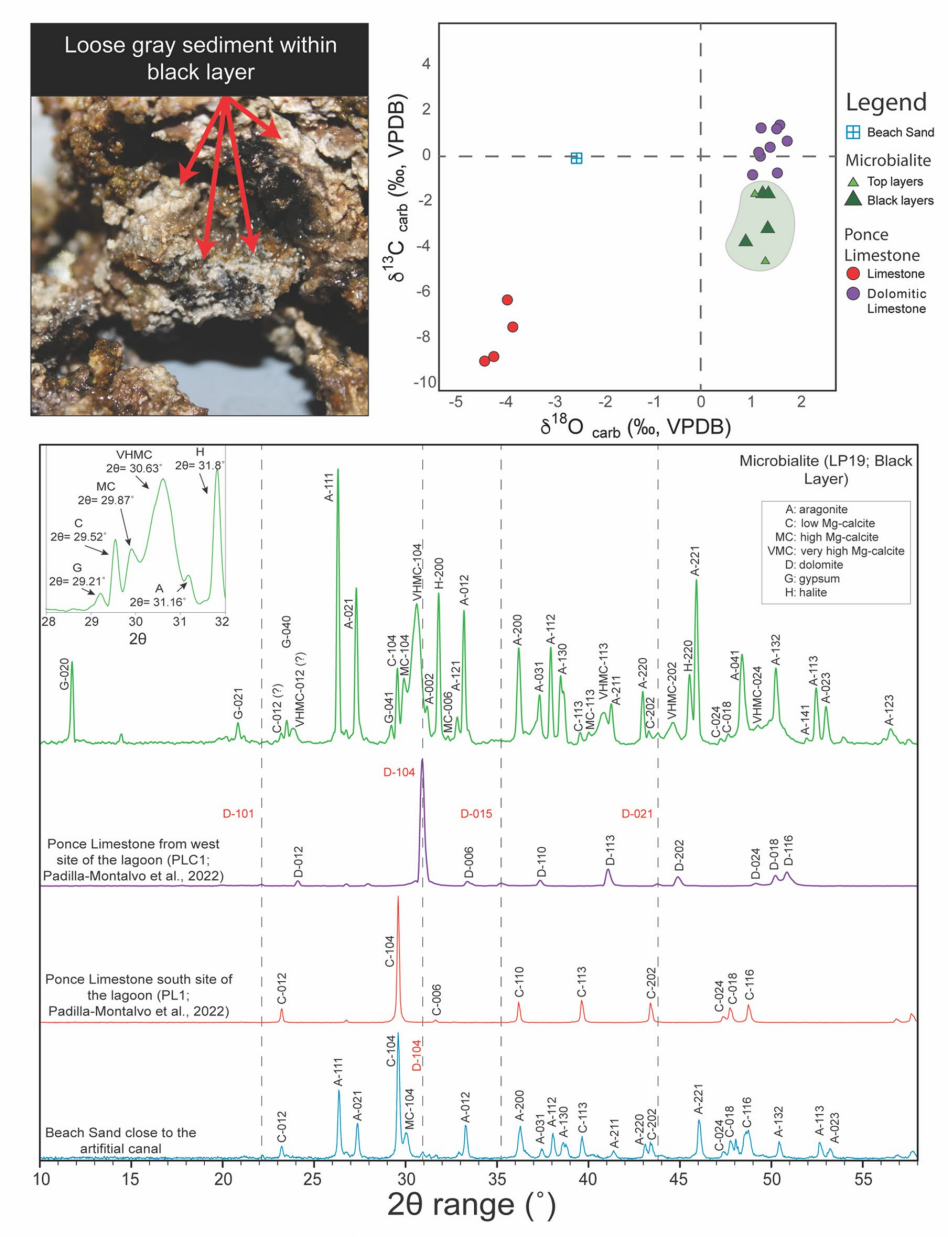
**Stable isotope and mineralogy for Mg-carbonate formation within microbialites from Laguna Providencia (LP). (Top left)** Field photograph of a microbialite showing the loose gray sediment hosted within the black layer, where Mg-enriched carbonate phases were sampled. **(Top right)** δ¹³C and δ¹⁸O values (‰ VPDB) of carbonates from microbialites (green symbols) compared with dolomitized and calcitic facies of the Ponce Limestone (purple and red circles)^26^, and beach sediment for reference (light blue cross-squares). **(Bottom)** Representative XRD patterns comparing the microbialite black layer (LP19) with Ponce Limestone and adjacent beach sand for reference. The microbialite contains abundant very high-Mg calcite (VHMC, or proto-dolomite) and aragonite (A), whereas the dolomitized Ponce Limestone shows ordered dolomite (D) reflections, shown across the dashed lines. Beach sand contained minerology coming from bioclastic sediment and lithoclasts from the Ponce Limestone. Miller indices (hkl) for the minerals were identified and included.

Spherulitic grains are abundant throughout the microbialite, occurring as small forms (ca. 10-25 μm) within microbial laminations in surface mats and as larger spherulites associated (ca. 250 μm) with EPS-rich zones and occasional bioclasts (Fig. 2c-d; Supplementary Fig. S5b-d online). Petrographic analyses show thrombolitic clots composed of spherulitic peloids, microspar, and acicular carbonate overgrowths (Fig. 2e), whereas stromatolitic intervals display irregular micrite-rich laminae (Fig. 2f). Fluorescence microscopy reveals strong signals in spherulite nuclei with weaker fluorescence along rims and overgrowths (Supplementary Fig. S5c-f online). Mineralogical staining further indicates pervasive Mg-calcite within spherulitic peloids and micritic laminae, with minor aragonite cements localized to rims and void-filling phases (Supplementary Fig. S5g online). SEM observations document spherulites with dense micritic nuclei and outward botryoidal aragonite growth, along with centric diatom valves, framboidal pyrite, putative amorphous Mg-silicates in deeper layers, and locally developed aragonite needles associated with organic matter (Fig. 3e-f; Supplementary Fig. S6c-d online) XRD analyses indicate a mineralogical progression from surface gypsum to aragonite and high-Mg calcite in intermediate layers, with the black organic-rich zones hosting the most diverse assemblages, including low-Mg calcite, high-Mg calcite, and very high-Mg calcite (VHMC; ca. 38.8 mol% MgCO₃), commonly associated with loosely consolidated gray sediment (Figs. 4c and 5).

## Stable Isotope Geochemistry

Carbonate δ¹³C values from LP19 range from -4.63‰ to -1.62‰, and δ¹⁸O values from + 0.9‰ to + 1.36‰ (Fig. 5; Supplementary Table S1 online). Dolomitic samples from nearby Ponce Limestone samples (Supplementary Fig. S7 online) display δ¹³C values between - 0.81‰ and + 1.38‰ and relatively enriched δ¹⁸O values from + 1.05‰ to + 1.74‰ (Supplementary Table S1 online). In contrast, calcitic Ponce Limestone samples show the most depleted compositions, with δ¹³C values from - 9.79‰ to -6.31‰ and δ¹⁸O values from - 4.33‰ to -3.77‰. Beach sediments exhibit δ¹³C of -0.08‰ and moderately depleted δ¹⁸O (-2.49‰).

## Microbial Community Composition

**Fig. 6:**
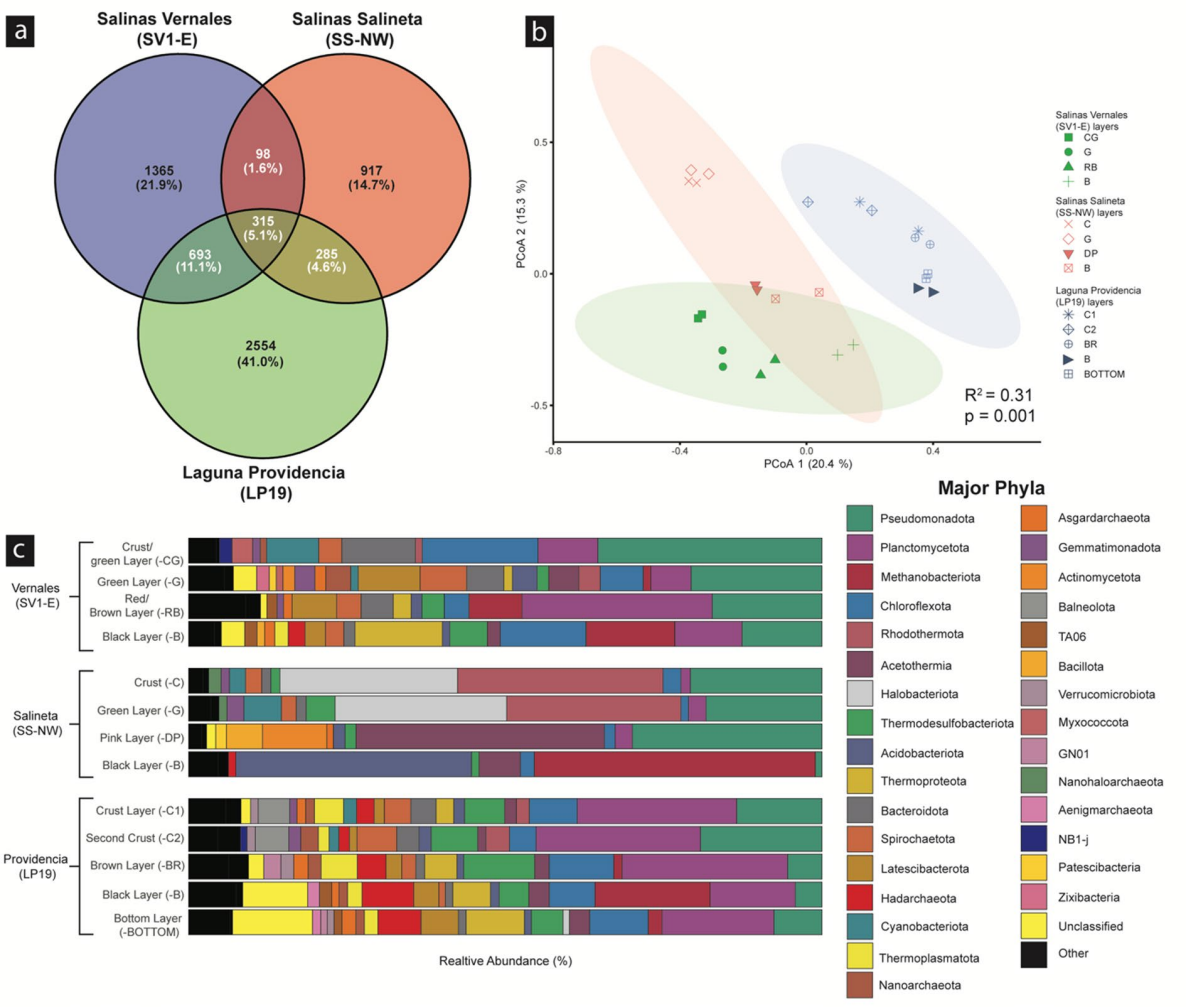
**Microbial community composition of microbial deposits from three hypersaline lagoon systems in Guánica**,** Puerto Rico: Salinas Vernales (SV1-E)**,** Salinas Salineta (SS-NW)**,** and Laguna Providencia (LP19). (a)** Venn diagram showing the distribution of unique and shared ASVs among the three sampled sites. Numbers indicate the count of ASVs detected exclusively in each site or shared across multiple sites. **(b)** Beta diversity Principal Coordinates Analysis (PCoA) of CSS-normalized microbial community composition across samples based on Bray-Curtis dissimilarity. Each point represents a sample colored by sample deposit and shaped by layers. Dashed ellipses show the approximate 95% confidence regions around the centroid of each site group, visualizing the dispersion and clustering of communities. PERMANOVA explains approximately 31% of the total variance in microbial community composition (Pseudo-F = 5.08, R² = 0.31, *p* = 0.001, 999 permutations), indicating significant compositional differences among lagoons. **(c)** Relative abundance of major microbial phyla across sample layers.

Microbial community composition differed among lagoons and mat layers, as reflected in both alpha- and beta-diversity analyses. Alpha diversity analyses revealed pronounced differences in richness and evenness across lagoons and mat layers (Supplementary Table S2). Microbialites from Laguna Providencia exhibited the highest diversity, with bottom and brown layers yielding over 1,200 observed ASVs and Shannon indices > 5.0, whereas the basal layer of Salinas Salineta showed markedly reduced diversity (Observed = 346.5 ± 313.25; Shannon = 2.55 ± 1.38). Surface layers from SV1-E and SS-NW displayed intermediate diversity (Shannon ca. 4.9). Venn analyses indicate substantial lagoon-specific ASV pools, with only 316 observed ASVs shared across all systems and limited pairwise overlap, particularly between SV1-E and SS-NW (Fig. 6a). Beta-diversity analyses reveal clear clustering by lagoon (Fig. 6b), with tight grouping of LP19 samples and distinct ordination space occupied by SV1-E and SS-NW. These differences are statistically significant (PERMANOVA: pseudo-F = 5.08, R² = 0.31, *p* = 0.001), indicating strong spatial structuring of microbial communities across lagoons.

*Salinas Vernales (SV1-E).* Bacterial taxa dominated upper mat layers (64.68-98.47%) and decreased with depth, whereas Archaeal taxa exhibited the opposite trend (1.53-35.33%; Supplementary Table S2). Surface crust and green layers were dominated by *Pseudomonadota* (20.67-35.38%), followed by *Chloroflexota* (order *Aggregatilineales;* 18.27-6.79%), *Latescibacterota* (9.78%), *Planctomycetota* (6.35-9.47%), *Bacteroidota* (5.87-11.62%), and *Spirochaetota* (3.65-7.36%) (Fig. 6c). Cyanobacteria (primarily *Desertifilaceae*) occurred in the crust (1.17-8.23%; Supplementary Fig. S8a online). *Thermodesulfobacteriota* ranged from 1.83% in the green layer to 5.93% in the black layer. Sulfate-reducing bacteria (SRB) included *Desulfomonilaceae* (in red layers), *Desulfosarcinaceae* (in black layer), and *Desulfohalobiaceae* (in both red and black layers; Supplementary Fig. S8c online). Archaeal taxa included *Nanoarchaeota* (up to 3.94%) and *Hadaarchaeota* (up to 2.66%). Methanogenic Archaea (*Methanobacteriota*), particularly unclassified *Methanofastidiosales*, peaked in the black layer (up to 14.03%; Supplementary Fig. S8d online).

*Salinas Salineta (SS-NW).* Microbial communities in SS-NW were dominated by Bacterial taxa (52.80-98.20%), but the microbial mats from this lagoon exhibited the highest relative abundance of Archaeal taxa (1.80-47.20%; Supplementary Table S2 online). *Pseudomonadota* and *Rhodothermota* dominated surface crust and green layers (18.31-20.77% and 27.50-32.44%, respectively; Fig. 6c). Cyanobacteria ranged from 2.56 to 5.91% and included *Rubidibacteraceae* and *Geitlerinemaceae* (Supplementary Fig. S8a online). Purple non-sulfur bacteria (PNSB; genus *Rhodovibrio*) were also abundant in crust and green layers (8.78-11.63%; Supplementary Fig. S8b online). Deeper pink and black layers were enriched in *Acetothermia* (6.54-39.23%), *Acidobacteriota* (37.24%), *Actinomycetota* (10.15%), and *Bacillota* (5.70%). Archaeal taxa were dominated by *Halobacteriota* (up to 28.07% in the crust), and methanogens, especially *Methanofastidiosales*, were more abundant here than in other lagoons, reaching 44.38% in the black layer (Supplementary Fig. S8d online).

*Laguna Providencia (LP19).* The microbialite in Laguna Providencia showed the highest overall diversity but lacked clear vertical stratification (Fig. 6c). Bacterial taxa decreased modestly with depth (84.66% to 64.69%), while Archaeal taxa increased (15.34-9.70% in crust to 24.10-44.98% at depth; Supplementary Table S2 online). *Planctomycetota* were consistently abundant (13.47-26.15%), *Pseudomonadota* were most represented in the crust (13.47-19.19%), whereas *Chloroflexota* (*Aggregatilineales*) was observed across all layers (2.13-10.19%). Cyanobacteria were present but low in abundance (1.52-2.03%), represented by *Rubidibacteraceae* (Supplementary Fig. S8a online). SRB included *Desulfohalobiaceae* (up to 6.33% in brown layers) and *Desulfatiglandaceae* (up to 3.06%; Supplementary Fig. S8c online). Archaeal taxa included *Nanoarchaeota* (1.38-2.76%), *Hadaarchaeota* (1.69-8.15%), and methanogenic taxa such as *Methanofastidiosales* (up to 18.18% in black layers; Supplementary Fig. S8d online).

## Discussion

The three lagoonal systems examined in this study define a clear continuum of microbial carbonate development, ranging from non-lithifying hypersaline mats at Salinas Salineta, through partially calcifying mats at Salinas Vernales, to fully lithified microbialites at Laguna Providencia. Although these lagoons occur within the same coastal region, they function as hydrologically independent basins rather than a single connected environmental gradient. While some datasets represent a single temporal snapshot, the combined sedimentological, petrographic, and microbial evidence indicates that lithification potential varies markedly over short spatial scales within a shared coastal setting. Across all systems, microbial mats develop on physically stable substrates, indicating that substrate availability influences deposit distribution; however, substrate stability alone is insufficient to explain carbonate lithification, as demonstrated by the non-lithifying mats developed on halite crusts in Salinas Salineta. Differences along the lithification continuum coincide with shifts in microbial community structure and diversity, which increase from Salinas Salineta to Salinas Vernales and are highest in the lithified microbialites of Laguna Providencia. However, these biological differences occur within distinct physicochemical environments, including variations in salinity across lagoons. These differences in community composition likely reflect environmental filtering associated with the distinct physicochemical conditions of each lagoon, whereby hypersalinity, pH, and hydrological stability may favor different microbial guilds associated with either non-lithifying or lithifying mat systems. Lithification therefore likely reflects the combined influence of microbial metabolism, environmental geochemistry, and system maturity, which together govern whether microbial mats remain unlithified, initiate calcification, or develop into lithified microbialites.

## Non-Lithifying Microbial Deposits in Salinas Salineta

The absence of lithification in microbial deposits from Salinas Salineta reflects strong geochemical and hydrological constraints that overwhelm biological influences during the observed conditions. In SS-NW, microbial mats are restricted to persistently submerged areas and colonize thick evaporitic crusts that dominate the benthic substrate (Supplementary Fig. S2b). High salinity and moderate alkalinity indicate a hydrologically restricted basin dominated by evaporative concentration (Table 1). Historical satellite and aerial imagery show that SS-NW frequently undergoes isolation and seasonal desiccation, and sampling near the end of the dry season (June 2019) likely captured conditions of intensified evaporation and gypsum-to-halite supersaturation. Under such conditions, carbonate dissolution likely exceeds precipitation, effectively preventing sustained lithification in this system.

Although microbial communities in SS-NW are well developed, their role in carbonate formation appears secondary to these physicochemical constraints. Surface layers are dominated by halophilic Archaea and PNSB (e.g., *Rhodovibrio sp.*), consistent with the observed pink pigmentation and a shift toward anoxygenic phototrophy under hypersaline conditions. Similar salinity-driven transitions toward anoxygenic phototrophy have been documented in other hypersaline microbial mats^31^. While Cyanobacteria are present, the dominance of anoxygenic phototrophs and sulfide-oxidizing taxa likely promotes net carbonate dissolution through CO₂ and proton release^32,33^. Minor occurrences of high-Mg calcite and aragonite in basal layers (Fig. 4b) may reflect localized, transient supersaturation, potentially linked to methanogenic activity, which can increase alkalinity by consuming organic acids and CO₂^34,35^. However, the absence of lithification across most of the Salinas Salineta system, including other basins, indicates that any carbonate precipitation is spatially restricted, short-lived, and insufficient to overcome basin-scale evaporative and geochemical controls.

## Calcifying Microbial Mats in Salinas Vernales

Unlike the non-lithifying mats at Salinas Salineta, SV1 hosts actively calcifying microbial mats under physicochemical conditions more favorable for carbonate precipitation. Field measurements indicate lower salinity and higher pH in SV1 relative to SV2, which exhibits geochemical characteristics more similar to Salinas Salineta (Table 1). These comparatively lower salinities may support more diverse and metabolically active microbial communities, including taxa and guilds directly involved in organomineralization, thereby enhancing lithification potential. Although short-term dilution from rainfall, surface runoff, or localized subsurface inputs may contribute to this contrast, the proximity of SV1 to the shoreline and lack of persistent freshwater surface inflow indicate a system fundamentally influenced by seawater. Over time, evaporation likely concentrates ions to levels permissive of carbonate supersaturation; however, the lower salinities observed at SV1 relative to SV2 suggest that additional hydrological inputs, potentially including subsurface water sources, warrant targeted geochemical and hydrological investigation.

Under these conditions, calcifying microbial mats preferentially colonize hard calcrete surfaces (Supplementary Fig. S1a-b online). The distribution of hemispheroidal mounds in central areas and polygonal mats along pond margins likely reflects gradients in water depth and exposure frequency (Supplementary Fig. S1a online), similar to patterns described for microbial mats in Little Ambergris Cay, Turks and Caicos^36^. Polygonal textures likely formed during episodes of shallow inundation and periodic exposure, whereas hemispheroidal mounds represent more mature structures developed under longer-lived submergence, although confirmation of this sequence will require targeted geomorphic and hydrological analyses.

Microscopically, SV1-E contains abundant micritic carbonate closely associated with EPS (Figs. 2a and 3a), including trigonal crystals comparable to those described from Lagoa Vermelha, Brazil^16^, Big Pond^17^, and Mérantaise River, France^37^. SEM imaging reveals small (ca. 5 μm) spherulites nucleating on or adjacent to microbial filaments (Fig. 3b), consistent with organomineralization either within porewaters or directly on EPS or filament surfaces^3^. Larger spherulites with micritic nuclei and sparry aragonite rims (Fig. 2b) resemble carbonate grains from other microbial calcifying systems (e.g.^16,17,38,39^) . The micritic nuclei likely reflect microbial micrite or partially micritized detrital particles incorporated during mat accretion. These observations indicate that carbonate production in SV1 is predominantly authigenic and microbially mediated or early diagenetic overgrowths, with minor contributions from detrital material, similar to textures documented in Abu Dhabi sabkha mats, where diagenetic aragonite rims overgrow earlier micritic nuclei, and in microbialites from the Great Salt Lake, USA, where organomineralization dominates early stages of precipitation^39^.

Microbial community patterns in SV1-E are consistent with a biogenic contribution to carbonate precipitation. Filamentous Cyanobacteria (represented in this case by *Desertifilaceae*) dominate the crust and green layers and are known to promote carbonate nucleation by generating EPS during photosynthetic CO₂ uptake^35^. Carbon fixation through photosynthesis can increase alkalinity, and at the same time, EPS acts as a trap for sediment particles and nucleation sites, provided that its cation-binding capacity does not inhibit mineral formation^3^. SEM imaging of filamentous structures within the green layer (Supplementary Fig. S6a online) provides visual support for their presence. Cyanobacteria are also well-documented contributors to microbial deposit morphotypes, and their elevated relative abundance in SV1-E compared to the other lagoons may help explain documented morphologies such as the observed pinnacle-like structures in some of the microbial mats (Supplementary Fig. S1e online). Deeper layers also host SRB (e.g., *Desulfohalobiaceae*, *Desulfomonilaceae*) and methanogens (e.g., *Methanofastidiosales*), whose metabolism of organic matter increases HCO₃⁻, further promoting carbonate supersaturation^35^. Although we cannot directly quantify the relative contributions of these guilds, the combined presence of filamentous Cyanobacteria, SRB, and methanogens suggests multiple metabolic and EPS-mediated pathways that likely contribute to the micritic and spherulitic textures observed in SV1.

### Microbialite Deposits in Laguna Providencia

Among the three studied lagoons, Laguna Providencia exhibits the most extensive lithified microbial deposits and, to our knowledge, represents the first formally documented microbialite buildup in Puerto Rico (Supplementary Fig. S3 online). This greater extent and degree of lithification likely reflect a longer period of microbialite development relative to Salinas Vernales and Salinas Salineta, as well as differences in present-day physicochemical conditions, a higher microbial community diversity, and local accommodation space. SfM-derived DEM data indicate that the western and northern sectors of the lagoon correspond to shallow, low-gradient zones consistent with laterally extensive microbialite accretion, whereas a steeper slope along the southern margin may have limited microbialite development in that direction (Supplementary Fig. S3a-i online). Similar slope-controlled zonation has been documented in lake microbialites from Laguna Pozo Bravo, Argentina^40^, and the Great Salt Lake^41^. Sedimentary records from Laguna Providencia (results not yet published) further indicate a complex paleoenvironmental evolution and a longer-lived lagoonal system, providing increased time for sustained microbialite accretion and maturation^42^. The central-lagoon position and localized distribution of the microbialite buildup, including meter-scale linear to curvilinear alignments, suggest a more complex hydrological history. Observations of low-salinity, high-alkalinity flows entering the lagoon from the Ponce Limestone (Table 1) raise the possibility of localized groundwater inputs or subtle tectonic influences associated with the nearby Punta Montalva fault^42^, although additional hydrological and geochemical data are required to evaluate these interpretations.

Internally, sub-fossilized microbialites in Laguna Providencia exhibit composite mesostructures, with stromatolitic textures at the base and thrombolitic fabrics toward the top (Supplementary Fig. S4 online). Such vertical changes in fabrics likely reflect temporal shifts in environmental conditions and microbial mat structure. Comparable composite microbialites have been described in modern systems, including Laguna Pozo Bravo^40^ and Shark Bay, Australia^43^, where fluctuating water levels, hydrodynamic energy, and microbial succession produce vertically stacked stromatolitic-thrombolitic sequences. In this case, the stromatolitic intervals were likely produced by filament-rich microbial mats capable of sediment trapping, binding, and in-situ organomineralization, similar to those observed in SV1. In contrast, thrombolitic textures appear consistent with coccoid-dominated pustular mats, such as those currently found at the surface of the microbialites. Genomic data support this interpretation, as pustular crust layers in LP19 show a higher relative abundance of coccoid cyanobacterial families such as *Rubidibacteraceae* compared to SV1-E, where filamentous cyanobacteria dominate. Shifts between filamentous and coccoid cyanobacteria have been linked to the formation of stromatolitic versus thrombolitic morphotypes in other lithifying mats^43,44^.

One point to consider is that Cyanobacteria occur at relatively low abundance in LP19 compared to other microbial groups, a pattern also observed in SV1-E and SS-NW, but to varying degrees. Similar discrepancies between observed mat morphologies and cyanobacterial sequence abundances have been reported in microbialite studies of Laguna Bacala, México^45^, and Storr’s Lake^46^. In these systems, cyanobacteria were also detected at relatively low sequence abundance despite their expected role in microbialite formation. These studies suggest that such discrepancies may reflect methodological factors in amplicon datasets, including primer bias, differential DNA extraction efficiency, and cell-wall-dependent lysis rates, although ecological differences in microbial community structure may also contribute. Although filamentous cyanobacterial morphologies are readily observed microscopically in SV1-E (Supplementary Fig. S6a online), reconciling the low cyanobacterial signal in LP19 will require targeted microscopy, taxonomic imaging, and possibly alternative molecular approaches. Another aspect to consider is the vertical structure of the sampled microbialite. Pustular mats occur only in the surficial crust, whereas deeper black microbial assemblages occur beneath a middle-lithified horizon. This configuration may explain the limited vertical stratification observed in the 16 S dataset (Fig. 6c) and suggests that the lithified substrate, rather than the actively growing microbial mat, exerts a strong influence on current community distribution. Additional spatial and seasonal sampling across the Laguna Providencia microbialite buildup will be needed to determine whether this pattern is consistent throughout the lagoon or reflects localized conditions at the sampled site.

Spherulites present within the pustular microbial mats associated with the thrombolitic microbialites (Fig. 2c-d) transition downward into clotted spherulitic textures in sub-fossilized sections (Fig. 2e; Supplementary Fig. S5c-d online), occasionally enclosing bioclasts (Supplementary Fig. S5b). These clotted fabrics, composed of spherulites and other unidentified peloidal grains embedded in acicular cements, closely resemble textures reported from microbialites in Lagoa Vermelha^16^, Rottnest Island in Western Australia^38^, Cuatro Cienagas in Mexico^47^, Lake Salda in Turkey^48^, and throughout the geologic record^1^. Microscopic examination of the deeper stromatolitic layers also revealed subtle sub-laminations composed of micritic and micropeloidal bands (Fig. 2f). The increased lithification within both stromatolitic and thrombolitic fabrics likely reflects microbial activity, potentially linked to EPS degradation following burial^17,49^, and diagenetic processes commonly associated with the alteration of porewaters during burial, which can promote early cementation^39,50^. In the stromatolitic intervals, these sub-laminations may reflect successive microbial biofilms, similar to those observed in SV1-E, that underwent progressive infilling by micritic precipitates. Within thrombolitic intervals, fluorescence microscopy and mineralogical staining together indicate organic-rich spherulite nuclei that likely served as initial nucleation sites for Mg-calcite micrite, followed by later aragonite precipitation forming less fluorescent rims and void-filling cements (Supplementary Fig. S5e-g)^16^. The development of these complex fabrics may therefore reflect the cumulative influence of a diverse, metabolically heterogeneous microbial community capable of sustaining multiple organomineralization and diagenetic pathways over time. These observations are in agreement with a paragenetic sequence in which Mg-calcite micrite precipitated first within organic-rich nuclei, where coccoid mats and later microbial activity during burial may have contributed to nucleation (e.g.^16,38^), and aragonite then precipitated during early diagenesis. The occurrence of framboidal pyrite within LP19 (Fig. 3f) could provide direct evidence of localized anoxia and active sulfur cycling, potentially linking sulfate-reduction activity, for example, to these paragenetic changes^16,51^. Although the contemporary microbial community may not represent the assemblages responsible for lithification, genomic data reveal significant populations of SRB, particularly *Desulfovermiculus* and *Desulfatiglans*, within the brown and black layers of LP19 relative to SV1-E and SS-NW (Supplementary Fig. S8c online).

### Putative Authigenic Mg-Carbonate Phases in Microbialites

The occurrence of VHMC confined to the black layer in LP19 is an intriguing result (Figs. 4c and 5). VHMC, together with high- and low-Mg calcite phases, was detected immediately beneath the middle-lithified crust within unlithified gray mud associated with the black-layer horizon and was not prominent in other LP19 microbialite layers (Fig. 4c). This stratigraphic confinement indicates that Mg-carbonate formation was restricted to a discrete microenvironment distinct from the overlying lithified fabrics. X-ray diffraction patterns show that this VHMC lacks distinct 101 and 015 reflections and exhibits only weak 021 ordering, consistent with poorly ordered proto-dolomite rather than fully ordered dolomite (Fig. 5)^52^. Although some ordering peaks may be obscured by aragonite signals, the Laguna Providencia microbialite VHMC remains mineralogically distinct from the ordered dolomite of the adjacent Ponce Limestone^26^.

Low-temperature dolomite formation remains a long-standing challenge in sedimentary geochemistry due to kinetic barriers to Mg incorporation under Earth-surface conditions (the “dolomite problem”)^53^. Increasing evidence from modern environments indicates that microbial activity (particularly sulfate reduction) can facilitate the formation of poorly ordered Mg-carbonate precursors that act as transient intermediates toward dolomite, as documented in microbial deposits from Lagoa Vermelha^54^, the Great Salt Lake^9^, the Abu Dhabi sabkhas^55^, and Petukhovskoe soda lake, Russia^15^. In LP19, the occurrence of VHMC within organic-rich gray sediment coincides with elevated SRB abundances relative to the other lagoons, consistent with microbially influenced organomineralization pathways reported in these systems. The presence of amorphous Mg-silicate phases within this horizon (Supplementary Fig. S6c online) suggests an additional formation pathway, whereby silicate- or clay-related precursors contribute to Mg incorporation and nucleation, in agreement with models from other microbial and evaporitic environments^55,56^.

Mineralogical, stratigraphic, and isotopic distinctions collectively support a predominantly authigenic origin for the VHMC (Fig. 5). Although both the microbialites and the Ponce Limestone exhibit relatively enriched δ¹⁸O values, carbonates from the Ponce Limestone record a multi-stage diagenetic history, with secondary dolomite and calcite displaying δ¹³C-δ¹⁸O compositions that remain isotopically distinct from the gray sediment hosting VHMC^26,57^. In contrast, depleted δ¹³C values in the microbialites are consistent with carbonate precipitation influenced by organic-matter degradation under reduced conditions^8,17^. Attempts to isolate the VHMC using dilute acetic acid resulted in dissolution, consistent with a poorly ordered and metastable Mg-carbonate rather than an ordered dolomite phase. Nevertheless, the isotopic signal likely reflects a mixed carbonate assemblage, and in the absence of porewater geochemistry or direct evidence of microbial nucleation, detrital input from Ponce Limestone dolomite cannot be excluded; accordingly, the VHMC mode of occurrence should be regarded as suggestive rather than conclusive.

### Concluding Remarks

This study documents the first known occurrence of actively lithifying microbial mats and microbialites in Puerto Rico, expanding the inventory of microbial carbonate systems within the Caribbean and providing new insight into why some microbial mats undergo lithification while others do not. The three lagoons examined represent a comparative continuum of microbial carbonate development within similar coastal settings, shaped by the interaction of microbial community composition with physicochemical and hydrological context. In Salinas Salineta, hypersaline and acidic conditions correspond to extensive but non-lithifying mats; in Salinas Vernales, more favorable conditions support active organomineralization, including micritic precipitation and spherulite development; and in Laguna Providencia, lithified microbialites preserve composite stromatolitic and thrombolitic fabrics and localized high-Mg carbonate phases indicative of microbially influenced early diagenesis. Together, these systems indicate that while substrate stability influences the spatial distribution of microbial deposits, sustained carbonate lithification is most closely associated with the development of diverse and compositionally distinct microbial communities operating within permissive hydrological and geochemical settings, potentially amplified over longer periods of system maturity.

Although porewater chemistry, metabolic rates, and temporal variability were not directly quantified, the close spatial coexistence of non-lithifying, partially lithifying, and fully lithified deposits within a small coastal region establishes the Guánica lagoons as a valuable natural laboratory for investigating microbial carbonate formation and biosignature preservation. In addition, the occurrence of poorly ordered Mg-carbonate phases within the Laguna Providencia microbialites bears directly on the low-temperature dolomite problem, indicating that microbialite-hosted environments can generate transient Mg-carbonate precursors distinct from detrital or diagenetic dolomite in adjacent carbonate units and providing a framework for distinguishing biogenic vs. abiotic signals in both terrestrial and putative extraterrestrial carbonate environments (e.g.^6^) . Given the uniqueness and vulnerability of these coastal systems, coordinated protective measures and educational initiatives are warranted to limit anthropogenic impacts. Responsible management of these lagoons is essential not only to safeguard a unique coastal ecosystem in Puerto Rico and the Caribbean, but also to preserve globally relevant analogs for understanding Earth’s early biosphere and the search for life beyond our planet.

### Methods

#### Fieldwork and Sample Collection

The microbial deposits described in this study were first documented during exploratory fieldwork in 2017, with initial observations focusing on the calcifying microbial mats from Salinas Vernales and non-lithifying microbial mats in the southeastern pond of Salinas Salineta (SS-SE). Subsequent fieldwork in 2019 and 2020 expanded documentation to include the other systems in Salinas Salineta (SS-C and SS-NW) and the microbialites in Laguna Providencia. The microbial community datasets presented here primarily derive from the 2019 fieldwork. All samples collected in 2019 were secured in sterile sampling bags, stored in a fridge during fieldwork, transported under sterile conditions to the Microbial Geochemistry Laboratory at the University of Kansas, and stored at -80 °C until further analysis. Samples were dissected and divided based on pigmentation differences across each microbial deposit. Microbialite buildup observations and the nomenclature of the microbialite structures were denominated following the terminology established by Grey and Awramik^58^. Ponce Limestone isotope data were added from Padilla-Montalvo^26^ (Supplementary Table S1 online), alongside additional Ponce Limestone and beach sand samples collected for this study. Water temperature, pH, conductivity, and salinity were measured directly in the field using a handheld multiparameter probe (AquaRead Ltd.). For alkalinity analyses, surface water samples were collected in the field, filtered through 0.20 μm syringe filters, and total alkalinity was determined by acid titration to a pH endpoint of 4.3 shortly after collection.

### Drone Imagery

Orthomosaic and multispectral drone imagery in Fig. 1c, f, i, Fig. S1a, Fig. S2a, and Fig. S3a-d were acquired over the three lagoon systems and surrounding landscapes during the 2019 field season. Visible-spectrum (RGB) imagery was collected using a DJI Phantom 4 Pro V2 unmanned aerial vehicle equipped with a 20-megapixel RGB camera (1-inch, 2.54 cm CMOS sensor), with flight missions designed to provide complete spatial coverage of each lagoon and adjacent areas relevant to hydrologic and geomorphic interpretation.

All flights were conducted using automated flight plans generated in DroneDeploy (version 2.202.0), incorporating a minimum of 80% forward overlap and 70% sidelap to ensure robust image matching and photogrammetric reconstruction. To minimize distortion associated with off-nadir viewing and refraction at the air-water interface, only the central, near-nadir portions of individual images were used during reconstruction.

RGB and multispectral imagery were processed into georeferenced orthomosaics (GeoTIFF format) using standard structure-from-motion (SfM) workflows in DroneDeploy and Agisoft Metashape Professional (version 2.0.1), including image alignment and point-cloud generation, with no major processing artifacts observed. A relative digital surface model (DSM) was derived from the RGB imagery, representing combined topographic and shallow bathymetric variability across lagoon basins and surrounding terrain. To facilitate qualitative interpretation of bathymetric and topographic gradients, the DSM was visualized using a false-color depth ramp, with warmer colors (red) indicating relatively shallower areas and cooler colors (green) indicating relatively deeper areas (Supplementary Fig. S4a-i).

### Petrography and X-Ray Diffraction

For petrographic analysis, select samples were impregnated with bio-epoxy and prepared as thin sections at the KU Department of Geology Rock and Thin Section Preparation Laboratory. Petrographic analysis was conducted using an Olympus BX53M optical microscope equipped with a motorized stage (Marzhauser Wezlar) and an X-Cite 120Q for fluorescence illumination. Microbialite fabrics and petrographic features were described following terminology established by Grey and Awramik^58^, and Flügel^59^.

Mineralogical characterization was performed across various depths. Each underwent treatment with 30% hydrogen peroxide (H_2_O_2_), air drying, and was ground for Powder X-ray diffraction analysis (PXRD). Analysis was performed using a Bruker D2 Phaser powder x-ray diffractometer (CoKᾱ radiation) equipped with a 1D mode Lynxeye detector. The analysis spanned a 2θ angle range of 5˚ to 90˚ at 0.03˚ increments every 0.3 s. A 24.6 mm × 1.0 mm zero-diffraction silica crystal plate (MTI Corp.) was inserted into the standard D2 Phaser sample discs and coated with grease to hold the powder. In some samples, Fischer Brand sodium chloride [NaCl] was added, and the halite [200] peak 2Q was used for peak alignment of calcite and dolomite [104] reflection displacements^60^. Scan results were processed (change to CuKᾱ spectra, background fitting, and alignment) and normalized for comparison using the R “powdR” package for XRD analysis^61^. Diffraction patterns were additionally inspected using the Jade XRD analysis software to verify peak assignments and Miller indices. The mole percentage of MgCO3 content in the Mg-carbonate precipitates was quantified using the Rietveld method (with the equation Mg% = 100 – (333.33x -911.99)^60^.

### Stable Isotope Geochemistry

Carbonate isotope geochemistry analyses were conducted at the Keck Paleoenvironmental Stable Isotope Laboratory at the University of Kansas. Samples underwent treatment with 30% H_2_O_2_, freeze-drying, and storage in low humidity before analysis. Each sample (75 µg) was meticulously weighed onto stainless steel boats using a Mettler Toledo microbalance. After an hour of vacuum roasting at 200 °C, samples were transferred to glass Kiel vials. Sample analysis involved reacting each sample with three drops of 100% H_3_PO_4_ at 70 °C for 540 s to generate CO_2(g)_ using a Thermo Scientific Kiel IV interfaced with the ThermoFinnigan MAT 253 dual inlet mass spectrometer. Both δ^13^C and δ^18^O data were calibrated against the VPDB scale using primary standards NBS-18 and/or NBS-19, with secondary standards (TSF-1, Sigma Calcite, and 88b Dolomite) employed for accuracy monitoring (with precision < 0.10‰).

### Scanning Electron Microscopy and EDS

Microbial mats and microbialite fragments were fixed in 2% paraformaldehyde in 0.1 M cacodylate buffer (pH 7.4) for 30 min, rinsed twice in buffer, and post-fixed in 1% osmium tetroxide for 1 h. Samples were dehydrated in a graded ethanol series (70%, 95%, and 100%) and dried using either critical point drying (CPD; Bal-Tec K850 critical point drier) or hexamethyldisilazane (HMDS) substitution. After drying, samples were mounted on 12.7 mm aluminum stubs with conductive silver paint. Some samples were sputter-coated with ca. 10 nm of iridium or gold using a Quorum 150RS sputter coater, while others were analyzed without coating. For uncoated preparations, variable pressure Scanning Electron Microscope (SEM) was used, or the samples were treated with Hitachi HILEM ionic liquid (diluted at 10-15%) to improve surface conductivity. In addition, selected microbialite samples from Laguna Providencia were examined directly from polished petrographic thin sections without coating.

Conventional imaging was performed with a Hitachi S-4700 cold field emission SEM, upper and lower secondary electron detectors, and an Oxford Instruments X-Max 150 EDX detector. Images were collected at 5-10 kV accelerating voltage, 5-10 µA emission current, condenser lens at 2, and 9-12 mm working distance. Energy-dispersive X-ray spectroscopy (EDS) was carried out at 10 kV using Aztec software (Oxford Instruments) to map the distribution of major elements within precipitates and biofilms. Variable pressure imaging was conducted with a Hitachi FlexSEM 1000 II SEM at 30 Pa chamber pressure, 5 kV accelerating voltage, and 10 mm working distance, also coupled to an Oxford EDX system. The critical point drier, the sputter coater, and the imaging on the S-4700 SEM/EDS were performed at the Microscopy and Analytical Imaging Research Resource Core Laboratory, University of Kansas. Imaging on the FlexSEM was performed at the Nanofabrication Facility, University of Kansas.

### DNA extraction, sequencing, and processing

Microbial DNA was extracted in duplicate from each depth interval using the Qiagen DNeasy PowerSoil kit following the manufacturer’s protocol. Extracted DNA was used for PCR amplification of prokaryotic communities (Bacteria and Archaea). Primers 515 F-Y^62^ and 806R-Y^63^ were used to target the V4 hypervariable region of the 16 S rRNA gene. PCR reactions included a mock community composed of bacterial and archaeal taxa in known concentrations as a quality control. Amplicon sizes were verified by gel electrophoresis. PCR products were purified using AmpureXP beads (Beckman Coulter), and dual-indexed libraries were prepared with Illumina Nextera XT v2 indices following the Illumina 16 S Metagenomic Sequencing Library Preparation protocol. The pooled libraries were sequenced on an Illumina MiSeq platform using a paired-end 300-cycle kit at the University of Kansas Genome Sequencing Core.

The raw sequencing data for this dataset were processed using Quantitative Insights into Microbial Ecology 2 (QIIME2–amplicon-2024.10)^64^. Demultiplexed reads were trimmed using the cutadapt plugin, where primer sequences were removed, and the DADA2 plugin was used to denoise and dereplicate sequences, infer ASVs, and filter chimeras. Taxonomy was assigned with the SILVA v138.2 reference database^65^, and sequences classified as chloroplasts and mitochondria were removed prior to downstream analyses. The data were normalized using cumulative-sum scaling (CSS) implemented in the R (v3.6.3) package metagenomeSeq^66^. The normalized dataset was further analyzed in R using the phyloseq package for community composition and diversity metrics, with additional analyses and visualizations performed using R packages ggplot2 and supporting packages such as dplyr and tidyr^67,68^.

Alpha diversity metrics, including Observed Species, Shannon, and Simpson indices, were calculated to assess microbial diversity using the estimate_richness function in the phyloseq R package^67^. To analyze beta diversity, Beta diversity was assessed using Bray-Curtis dissimilarities calculated from normalized abundance data in phyloseq. Principal Coordinates Analysis (PCoA) was performed with the ordinate function, and ordination plots were generated in ggplot2^67,68^. Differences in microbial community composition across lagoons and layers were statistically evaluated using Permutational Multivariate Analysis of Variance (PERMANOVA) in the vegan package^69^. PERMANOVA was conducted with the adonis2 function, using Bray-Curtis dissimilarities as the response variable and sample grouping as the explanatory variable, with 999 permutations. Results are reported as the proportion of variance explained (R²) and associated p-values. Venn diagrams were constructed with the ggvenn package and were based on the number of shared and unique amplicon sequence variants (ASVs) to visualize overlap in microbial community composition among lagoons and depositional layers^70^.

## Supplementary Information

Below is the link to the electronic supplementary material.

Supplementary Material 1

## Data Availability

Raw 16 S amplicon sequencing data are available on Figshare^71^ . All bioinformatics and statistical analysis scripts used in this study are openly available on GitHub at https://github.com/BryanRodriColon/Geobio_Guanica_Codes. All remaining data supporting the findings of this study are included within the manuscript and its Supplementary Information. The sUAS imagery is available upon request.
