# Supplementary material for: Documenting the occurrence of microbial carbonate deposits in Puerto Rico
Source: Sci Rep. 2026 Apr 13;16:19672. doi: 10.1038/s41598-026-47709-x (PMC13315269; doi:10.1038/s41598-026-47709-x)
Supplement: Supplementary file 1 — Supplementary Material 1 [file 41598_2026_47709_MOESM1_ESM.pdf]

Supplementary Information for the Manuscript *"Documenting the Occurrence of Microbial Carbonate Deposits in Puerto Rico"*

by

Bryan J. Rodríguez-Colón[1,*], Wilson R. Ramírez-Martínez[2], Aliyah M. Chabrier-Alpi[3], Carlos Ríos-Velázquez[4], Noraida Martínez-Rivera[5], Gregory S. Baker[6], Yasawantha Hiripitiyage[7], Belinda S.M. Sturm[7], Jennifer A. Roberts[1]

[1]Department of Geology, University of Kansas, Lawrence, Kansas 66045, USA.

[2]Department of Geology, University of Puerto Rico–Mayagüez, Mayagüez, Puerto Rico 00681, USA.

[3]Department of Marine Sciences, University of Puerto Rico–Mayagüez, Mayagüez, Puerto Rico 00681, USA.

[4]Department of Biology, University of Puerto Rico–Mayagüez, Mayagüez, Puerto Rico 00681, USA.

[5]Microscopy and Analytical Imaging Laboratory, University of Kansas, Lawrence, Kansas 66045, USA.

[6]Geoscience Program, Colorado Mesa University, Grand Junction, CO, 81501 USA.

[7]Department of Civil, Environmental & Architectural Engineering, University of Kansas, Lawrence, Kansas 66045, USA.

*bryan.rodriguez@ku.edu

Submitted to *Scientific Reports*

Supplementary Tables:

Sample	$\delta^{13}C_{carb}$ VPDB	$\delta^{18}O_{carb}$ VPDB
Microbialite_LP19-Crust	-4.63	1.3
Microbialite_LP19-MiddleCrust	-1.68	1.08
Microbialite_PL19-BlackLayer1	-1.62	1.25
Microbialite_PL19-BlackLayer2	-3.16	1.34
Microbialite_PL19-BottomLayer1	-1.63	1.36
Microbialite_PL19-Bottomlayer2	-3.74	0.9
PL-13A	-7.5	-3.77
PL-13B	-6.31	-3.87
PL-1A	-8.8	-4.15
PL-1B	-9	-4.33
PL-Outcrop_1m*	1.25	1.21
PL-Outcrop_6m*	0.01	1.22
PL-Outcrop_10m*	-0.73	1.54
PL-Outcrop_12m*	0.18	1.17
PL-Outcrop_23m*	1.38	1.58
PL-Core_1m*	0.41	1.38
PL-Core_8m*	1.24	1.53
PL-Core_6m*	0.72	1.74
PL-Core_13m*	-0.81	1.05
PL-BeachSand	-0.08	-2.49

Table S1. Stable isotope composition of carbonate samples from Laguna Providencia and surrounding units. Carbon ($\delta^{13}C$) and oxygen ($\delta^{18}O$) isotope values for microbialite carbonates from Laguna Providencia, along with comparative values from Ponce Limestone bedrock and modern beach sand samples. Samples marked with an asterisk (*) were previously reported by Padilla-Montalvo[1].

Lagoon and sample name	Sample Layer	Sample Layer	Observed	Shannon	Simpson	Bacteria Relative Abundance (%)	Archaea Relative Abundance (%)
**Vernales (SV1-E)**	C/G	SVE1-CG	426.5 ± 55.86	4.05 ± 0.19	0.95 ± 0.01	98.47	1.53
	G	SVE1-G	752.5 ± 12.02	4.95 ± 0.27	0.97 ± 0.02	90.41	9.59
	R	SVE1-R	775.5 ± 228.4	4.79 ± 0.26	0.97	85.08	14.92
	B	SVE1-B	914 ± 56.57	5.14 ± 0.07	0.98	64.67	35.33
**Salineta (SS-NW)**	C	SS-C	245.5 ± 118.09	3.97 ± 0.19	0.94 ± 0.04	69.09	30.91
	G	SS-G	686.5 ± 16.26	4.88 ± 0.21	0.97	70.35	29.65
	DP	SS-DP	58 ± 41.01	2.56 ± 0.15	0.84 ± 0.06	98.20	1.80
	B	SS-B	346.5 ± 313.25	2.55 ± 1.38	0.75 ± 0.16	52.80	47.20
**Providencia (LP)**	C1	LP-C1	1182 ± 11.31	5.53 ± 0.48	0.98 ± 0.02	84.66	15.34
	C2	LP-C2	993 ± 210.72	5.23 ± 0.34	0.98 ± 0.01	90.30	9.70
	BR	LP-BR	1278.5 ± 200.11	5.5 ± 0.27	0.99	75.90	24.10
	B	LP-B	1074 ± 148.49	5.17 ± 0.17	0.96 ± 0.03	55.02	44.98
	BOTTOM	LP-BOTTOM	142 ± 53.03	5.57 ± 0.05	0.99	64.69	35.31

Table S2. Alpha diversity metrics of microbial communities across samples with domain-level percentages. Values shown include observed ASV richness, Shannon diversity index, and Simpson diversity index, where observed richness represents the number of unique ASVs detected and the Shannon and Simpson indices describe community diversity and evenness. Percentages of reads assigned to Archaea and Bacteria are also reported for each layer. Sample-layer abbreviations: C, crust; G, green layer; R, pink layer; B, black layer; DP, dark pink layer; BR, brown layer; BOTTOM, basal microbialite layer.

Supplementary Figures

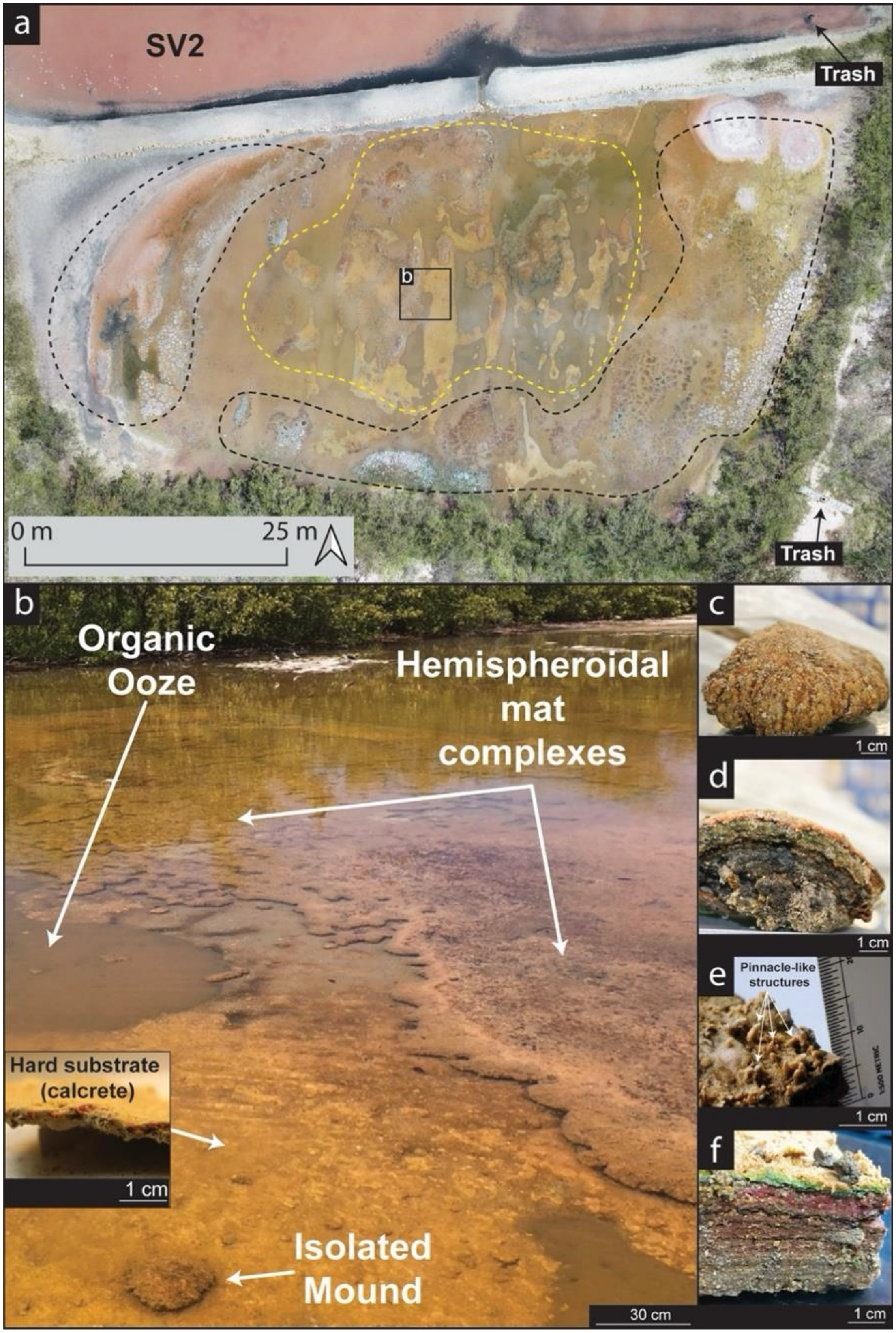

Figure S1. Field overview and sample context of calcifying microbial mats at Salinas Vernales (SV), southwestern Puerto Rico. (a) Drone image of pond SV2 showing distinct surface morphologies. The yellow dashed line outlines areas with hemispheroidal mat complexes, while black dashed lines indicate polygonal microbial mats and evaporitic crusts. **(b)** Field photograph of SV1 showing hemispheroidal mat complexes and isolated mounds colonizing calcrete substrates. The inset highlights the calcrete hardground overlain by a laminated microbial mat. **(c-f)** Representative mat morphologies and vertical structures, including **(c)** Hemispheriodal domes, **(d)** cross-section showing distinct organic-rich and carbonate laminae across different pigmentations, **(e)** pinnacle-like surface morphologies across flat microbial mats, and **(f)** dissected flat mat sample displaying color-banded layering from surface crust to basal sections. *Panel (a) corresponds to drone-derived orthomosaic imagery (see Methods for acquisition and processing details). The image in panel (b) was taken by Gregory Baker. Images in panels (c-f) were taken by Bryan Rodríguez-Colón.*

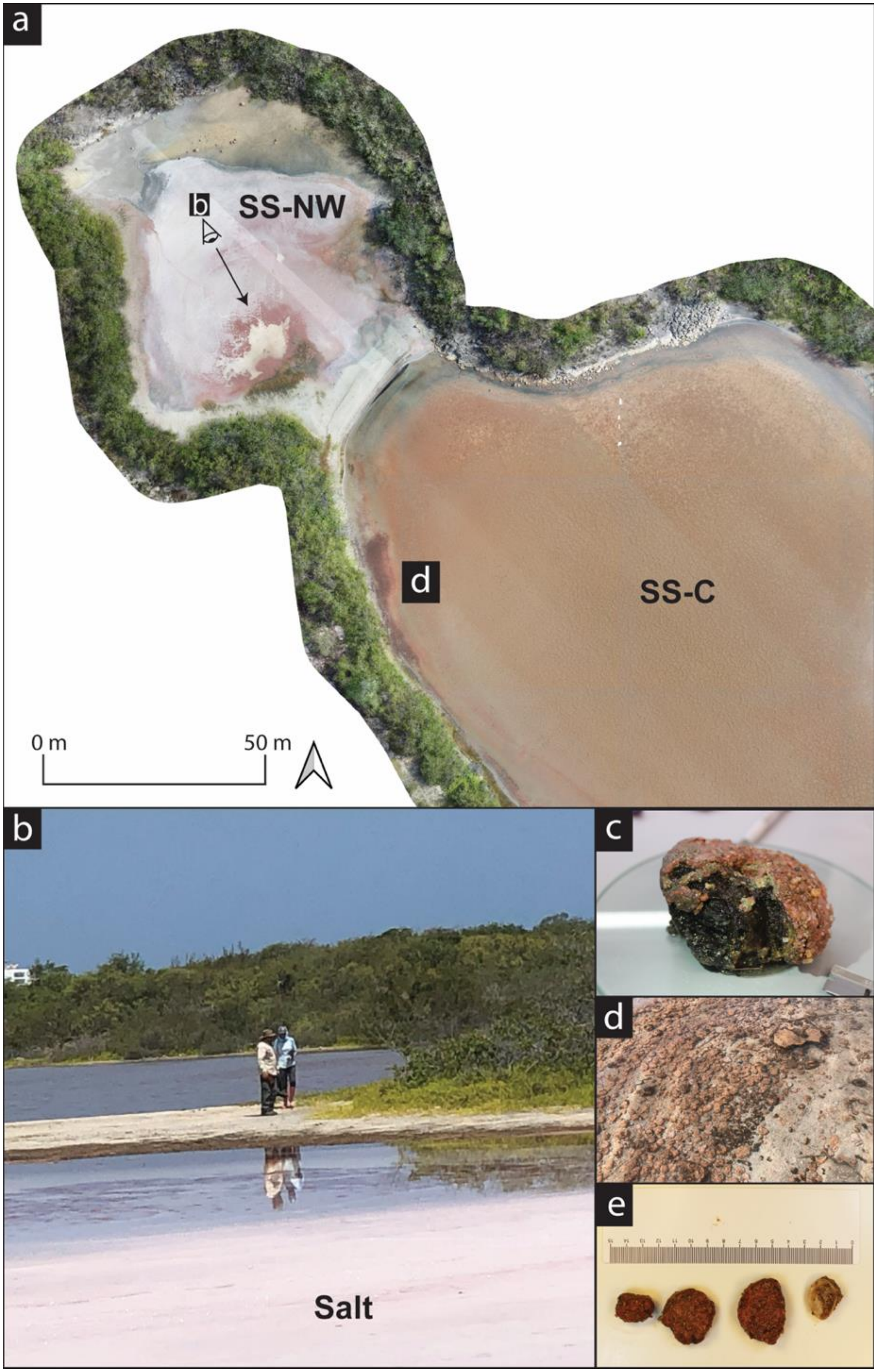
a
b
SS-NW
d
SS-C
0 m
50 m
b
Salt
c
d
e

Figure S2. Field overview and sample context of non-lithifying microbial mats at Salinas Salineta (SS), southwestern Puerto Rico. (a) Drone image from fieldwork 2019 campaign showing the northwest pond (SS-NW) and central pond (SS-C). The pink and white surface coloration corresponds to halite and gypsum crusts associated with desiccation and evaporite formation. **(b)** Field photograph of the SS-NW pond margin as observed from the northern area showing the extensive salt crust and shallow water conditions during sampling. **(c)** Representative microbial mat fragment displaying dark organic-rich material overlain by a thin halite crust. **(d-e)** Close-up in SS-C showing microbial mat chips growing in bioclasts (ca. 1-2 cm diameter) observed in the field. *Panel (a) corresponds to drone-derived orthomosaic imagery (see Methods for acquisition and processing details). Images in panels (b-e) were taken by Bryan Rodríguez-Colón.*

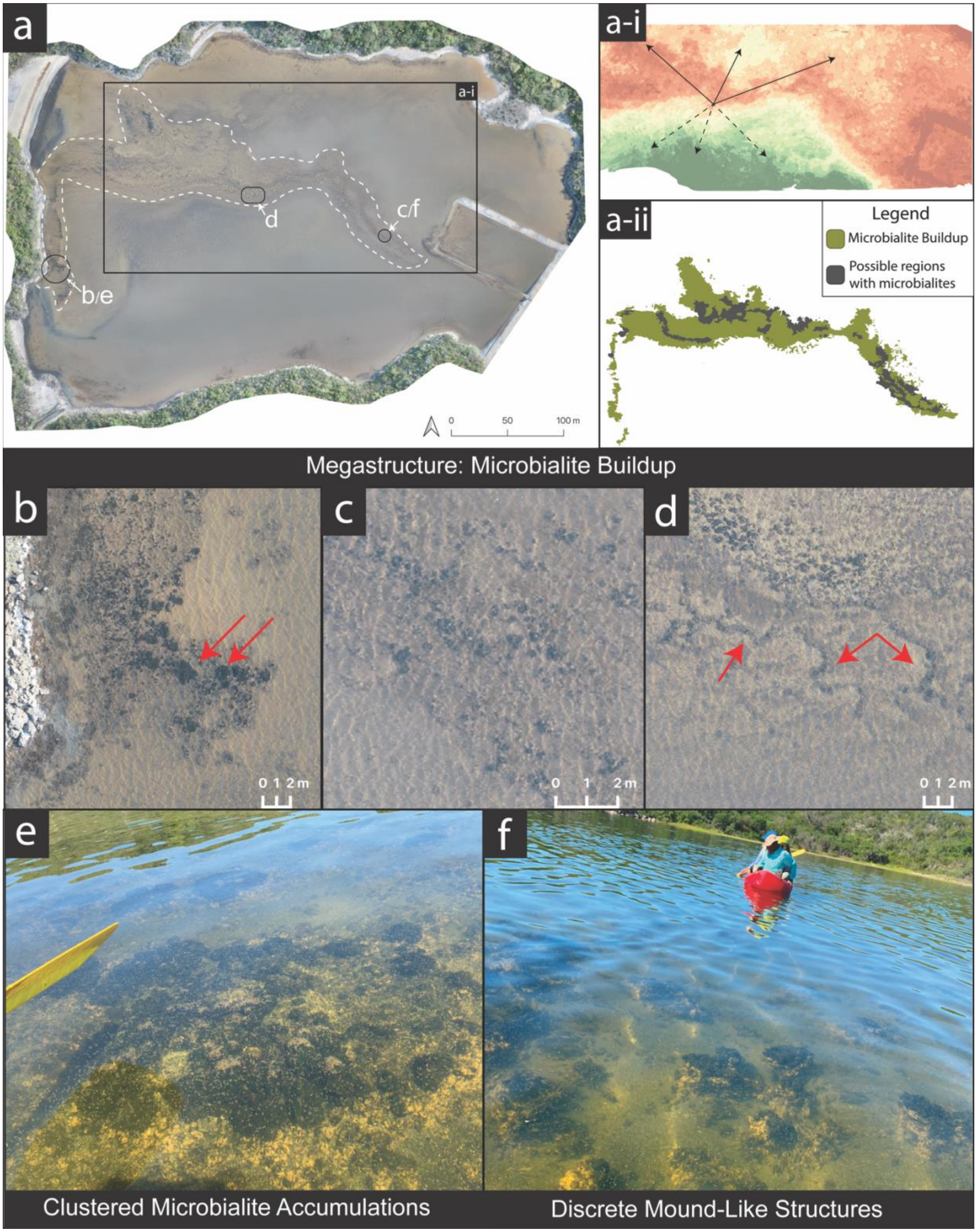

Figure S3. Field overview of microbialite buildup megastructure at Laguna Providencia (LP), southwestern Puerto Rico. (a) Drone image from 2019 fieldwork campaign showing the

spatial extent of the microbialite megastructure across the lagoon's central sector. White dashed lines outline the main microbialite buildup. **(a-i)** Bathymetric model illustrating the morphology of the microbialite structure; dashed arrows indicate steep slopes (green area), whereas solid arrows denote gentler gradients (red area). **(a-ii)** Vectorized image of the microbialite buildup distribution, highlighting the areas where microbialites are most likely concentrated (green color), with areas of either no microbialites present or buried microbialites (dark gray color). **(b)** Drone image showing clustered microbialite accumulations forming laterally continuous patches. **(c)** Drone image showing discrete, isolated mound-like microbialite structures. **(d)** Drone image showing linear to elongate arrangements of microbialite heads, forming meter-scale alignments. **(e)** Field photograph taken in January 2020 corresponding to the clustered microbialite accumulations shown in (b). **(f)** Field photograph corresponding to the isolated microbialite structures shown in (c). *Panel (a-d) corresponds to drone-derived orthomosaic imagery (see Methods for acquisition and processing details). Images in panels (e-f) were taken by Wilson Ramírez-Martínez*

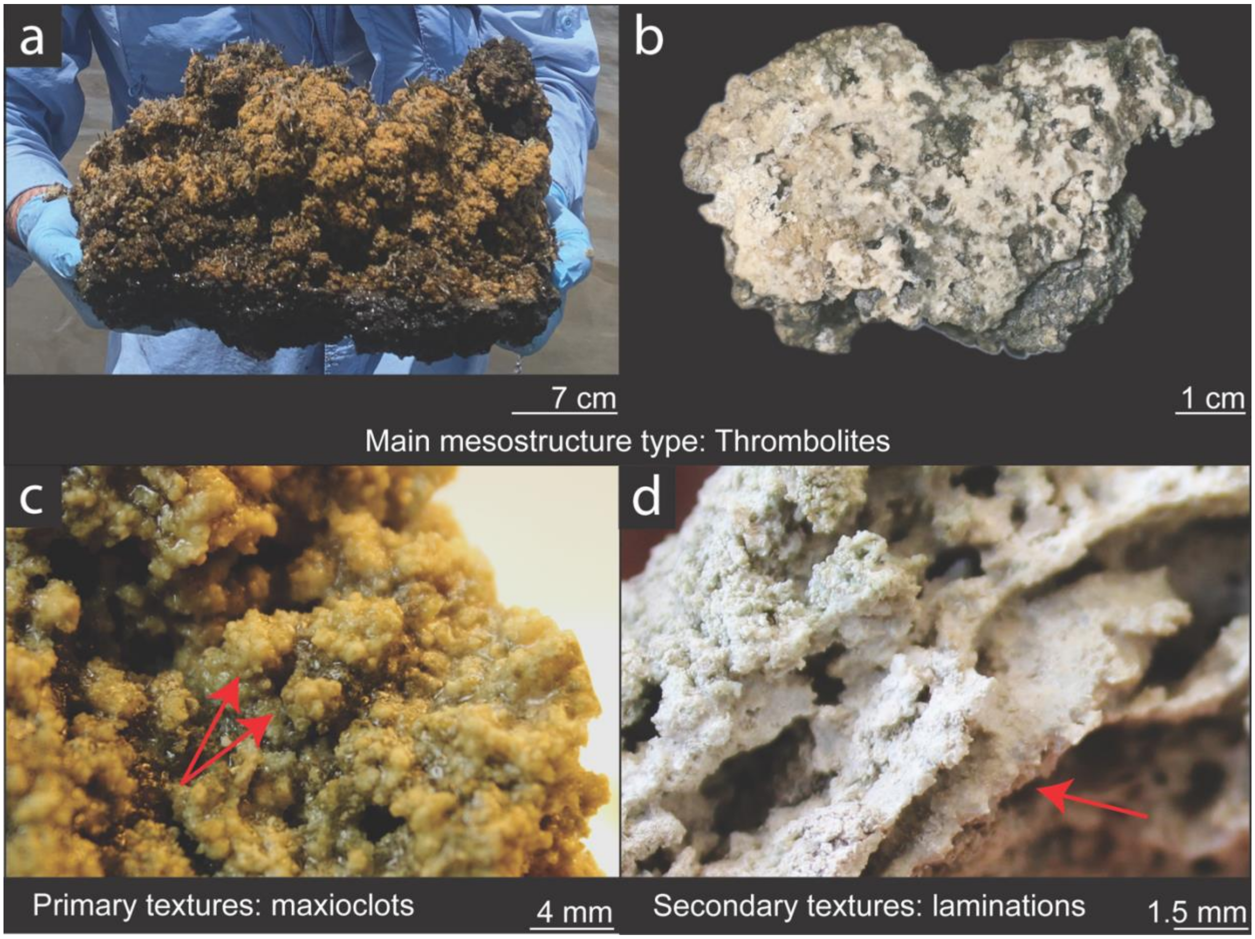

Figure S4. Macro- and meso-scale structure of microbialite buildups at Laguna Providencia (LP), southwestern Puerto Rico. (a) Representative microbialite from the western sector of the lagoon showing irregular clotted macrofabrics and an actively growing pustular microbial mat at the surface. **(b)** Sub-fossilized portions of the microbialite dominated by clotted internal textures. **(c-d)** Close-up views illustrating the composite structure of LP microbialites, including (c) primary clotted fabrics and (d) secondary irregular laminated textures locally intergrown within the deposit. *The image in panel (a) was taken by Gregory Baker, and the images in panels (b-d) were taken by Bryan Rodríguez-Colón.*

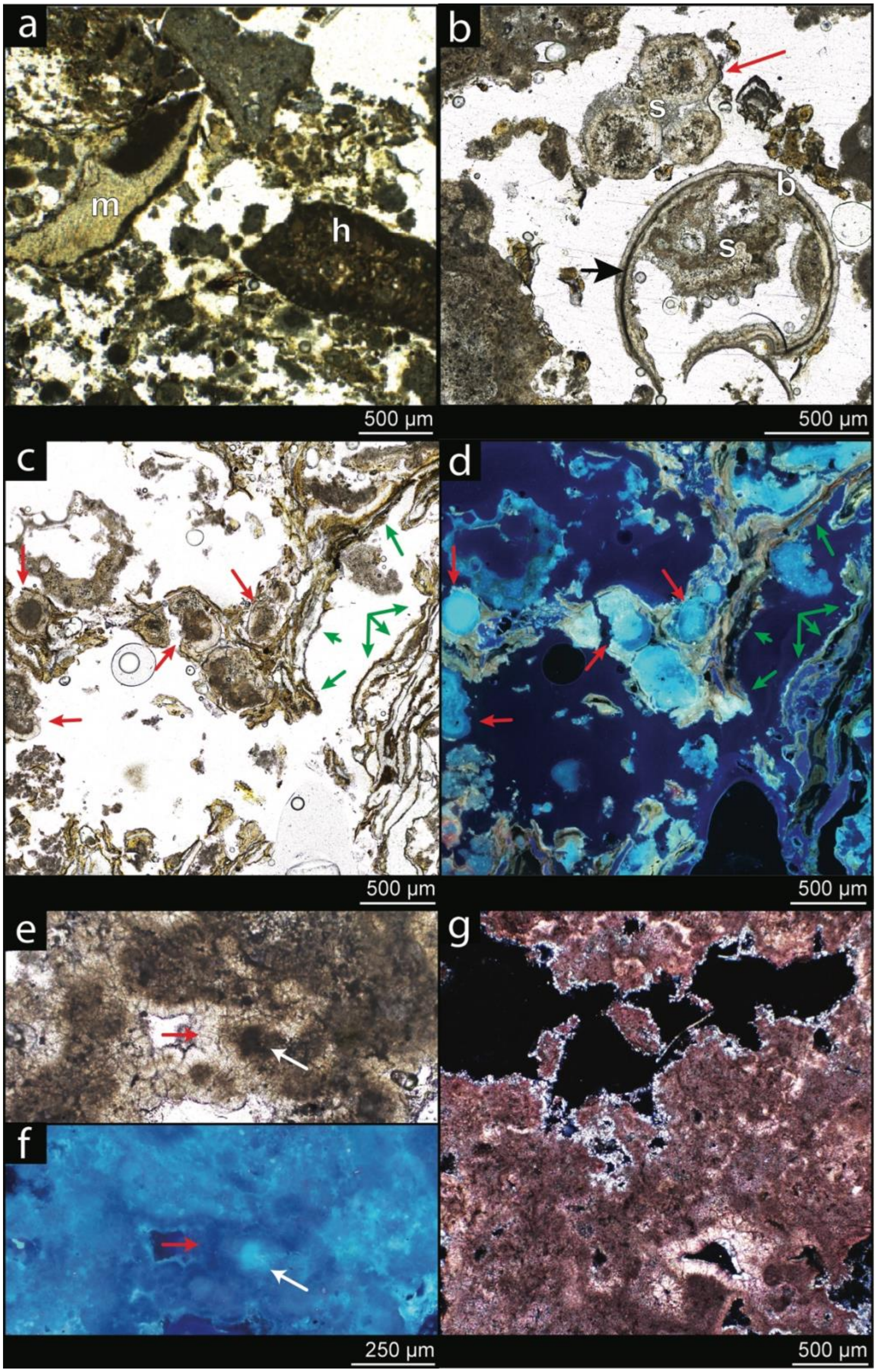

Figure S5. Additional petrographic characteristics of microbialite and microbial mat samples from Salinas Vernales (SV1-E) and Laguna Providencia (LP19), southwestern Puerto Rico. (a) Thin section from SV1-E showing bioclastic fragments of mollusks (letter m)

and *Halimeda spp.* (letter h) embedded in micritic matrix, indicating minor detrital input within an otherwise authigenic framework. **(b)** Microbialite thin section from LP19 showing composite spherulites (letter s) associated with micritic envelopes and bivalve fragments (letter b). The bivalve fragment hosts internally clustered spherulites, suggesting *in-situ* precipitation within pore microenvironments. **(c-d)** Active microbial mats from LP19 showing biofilm laminae containing spherulitic aggregates. **(c)** Plane-polarized light reveals microbial laminations (green arrows) and embedded spherulites (red arrows). **(d)** The corresponding fluorescence image highlights enhanced luminescence within spherulite centers. **(e-f)** Sub-fossilized thrombolitic section from LP19 under **(e)** plane-polarized and fluorescence **(f)** light. Fluorescence is concentrated at the centers of clustered spherulites (white arrow). **(g)** Thin section of LP19 microbialite stained with Alizarin Red S and potassium ferrocyanide, showing widespread Mg-calcite (purple areas) with minor aragonite (light pink areas) infilling voids, indicative of possible early diagenetic recrystallization.

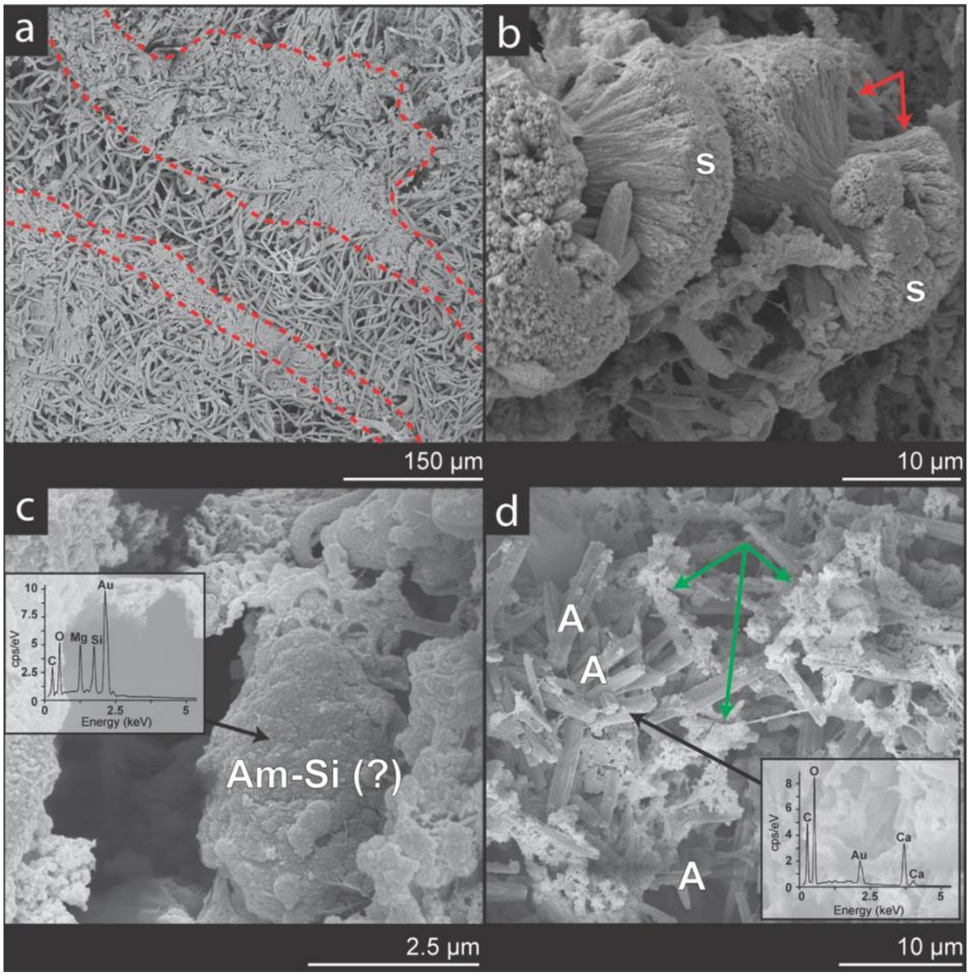

Figure S6. Additional Scanning Electron micrographs of microbial fabrics and mineral phases from Salinas Vernales (SV1-E) and Laguna Providencia (LP19), southwestern Puerto Rico. **(a)** Overview of SV1-E sample showing intertwined microbial filaments and micritic layers (red dashed areas). **(b)** Detail of SV1-E spherulites (letter s) forming dumbbell-shaped aggregates with fibrous internal textures (blue arrows). **(c)** SEM image of LP19 showing a putative amorphous Mg-silicate phase (letters Am-Si), confirmed by corresponding Energy Dispersive X-ray Spectroscopy (EDS) inset highlighting Mg and Si peaks. **(d)** SEM image from the basal section of LP microbialites showing randomly oriented aragonite needles (letter A, confirmed with EDS with a lack of Mg), surrounded by organic matter (green arrows).

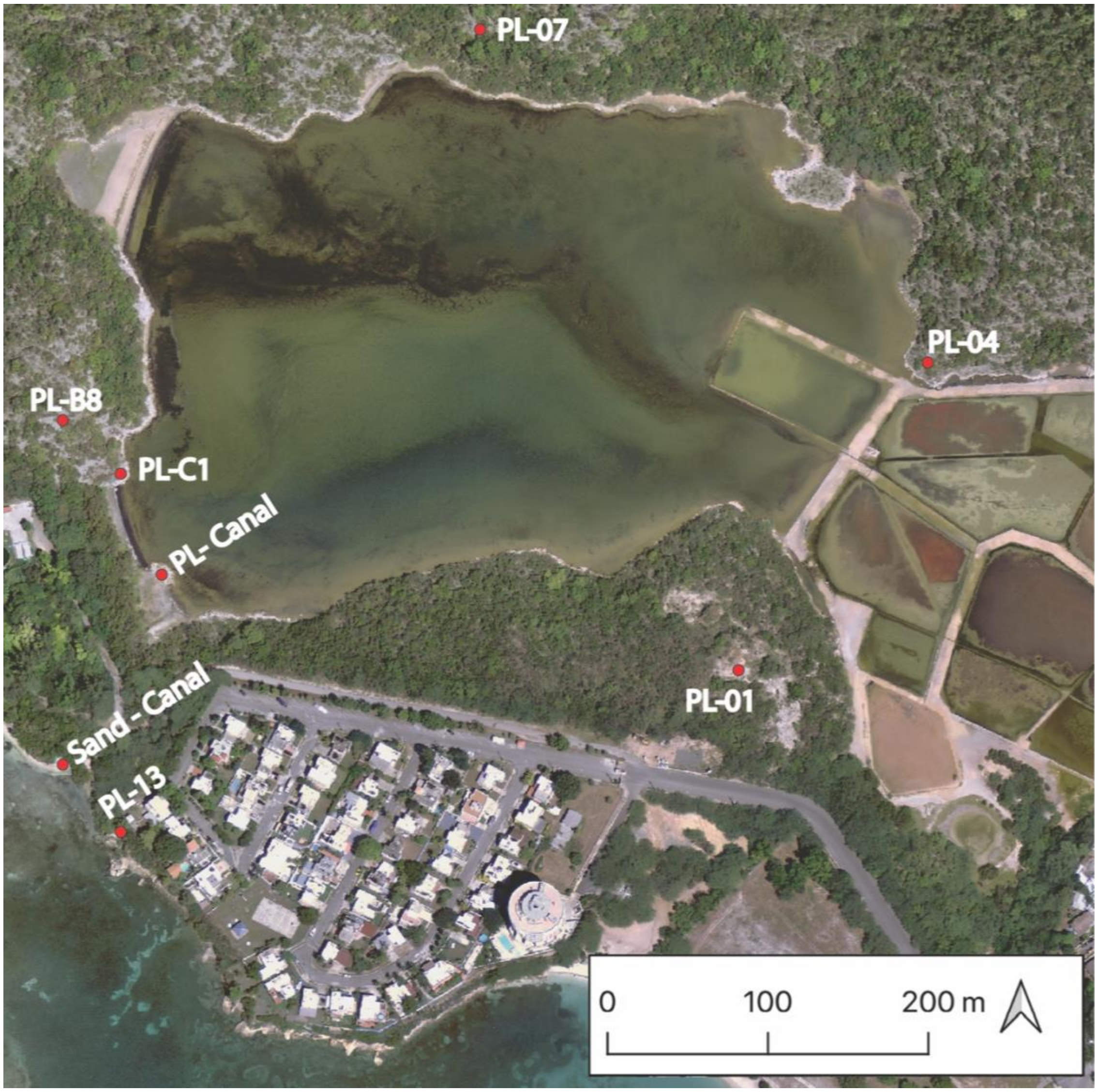

Figure S7. Sampling locations around Laguna Providencia, southwestern Puerto Rico. Map showing the locations where Ponce Limestone bedrock and modern beach sand samples were collected around Laguna Providencia for comparative mineralogical and stable isotope analyses[1]. The microbialite buildup is visible across the central portion of the lagoon in the 2010 orthomosaic image. *Orthomosaic imagery is derived from USGS EROS High Resolution Orthoimagery (HRO), which is publicly available and in the public domain.*[2]

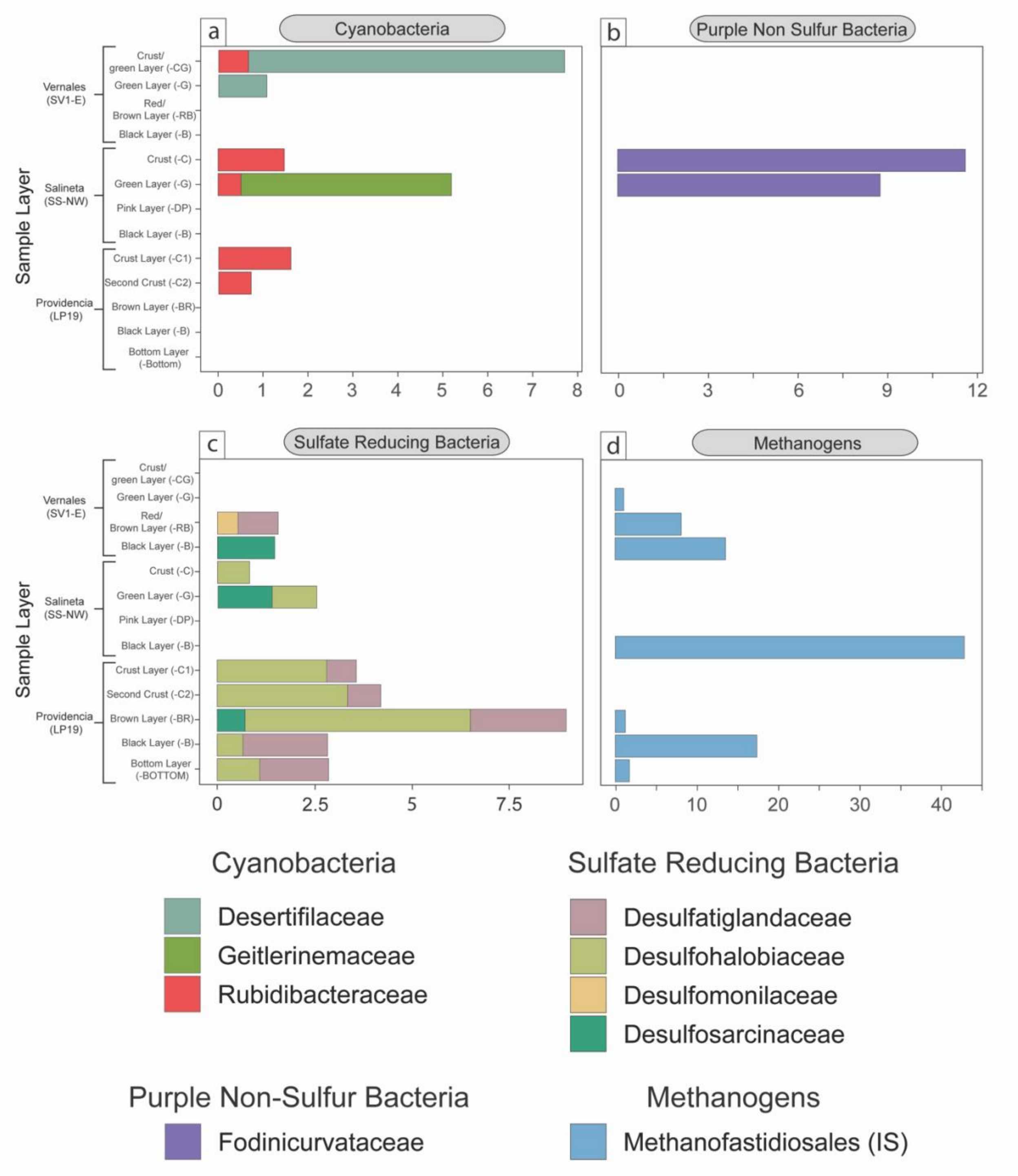

Figure S8. Relative abundance of major microbial guilds across microbial mat and microbialite layers. Bar plots show the distribution of key microbial groups discussed, such as Cyanobacteria, Purple Non-Sulfur Bacteria, Sulfate-Reducing Bacteria, and Methanogens. IS means Incertae Sedis. Taxa with relative abundance ≤0.5% were omitted from guild plots for visualization clarity.

References

1 Padilla-Montalvo, J. A., Ramirez-Martinez, W. R., Rodriguez-Colon, B. J. & Roberts, J. A. Petrography and Geochemistry of Dolomites in Montalva, Guánica Tools to Assess the Timing and Process of Dolomitization. Geological Society of America *Abstracts with Programs* 54, 5, Abstract 144-144 (2022). 10.1130/abs/2022AM-383098

2 U.S. Geological Survey. USGS EROS Archive: Aerial Photography & High-Resolution Orthoimagery (HRO) (U.S. Geological Survey). https://doi.org/10.5066/F73X84W6 (accessed September 20, 2024).
